# Model-based exploration is measurable across tasks but not linked to personality and psychiatric assessments

**DOI:** 10.1038/s41598-025-09152-2

**Published:** 2025-07-28

**Authors:** Kristin Witte, Mirko Thalmann, Eric Schulz

**Affiliations:** Center for Computational Health, Helmholtz Munich, 85764 Neuherberg, Germany

**Keywords:** Exploration–exploitation, Exploration strategies, Few-armed bandits, Reliability, Validity, Human behaviour, Statistics

## Abstract

An increasing number of studies have used multi-armed bandit tasks to investigate individual differences in exploration behavior. However, the psychometric properties of exploration measures remain unexplored. We examine the test–retest reliability, convergent, divergent, and external validity of model-based estimates of exploration strategies using three canonical paradigms. Our results revealed poor to moderate reliability, with minimal correlations for the same strategy across tasks. We then provide actionable recommendations for how to improve reliability and convergence across tasks: Simplifying common computational models enabled us to identify two convergently valid latent factors representing value-guided and directed exploration. Still, these factors showed neither a significant correlation with self-reported exploration tendencies nor with mood fluctuations, symptoms of anxiety, and depression. The exploration factors were, however, highly correlated with working memory capacity, questioning whether they provide additional information beyond performance-related constructs. To improve future research, we suggest simplifying common computational models and using multiple tasks to more accurately measure exploration strategies and mitigate spurious correlations arising from task-specific factors.

## Introduction

To explore or not to explore has significant implications. For many organisms, exploration is key to discovering new options. In humans, this might be akin to joining a new social group or trying out a new sport. The unfamiliar option could turn out worse than the one already known, but it might also prove better. Exploitation, on the other hand, involves selecting the option that one knows already to be good. The balance between exploration and exploitation is essential, and has been examined in a wide range of domains (for a review see Mehlhorn et al.^1^). With regards to human behavior, one question has been of chief interest in the past two decades: What are the cognitive strategies people apply to explore and exploit their environment?

Exploration strategies have been typically studied in so-called few-armed bandit tasks. In these tasks, participants are instructed to maximize their rewards by sequentially selecting between two to four response options (i.e., the bandits’ arms). At the onset of each round, participants must learn which arm yields the highest average reward, thereby creating an exploration-exploitation dilemma. Rewards on the arms are usually randomly sampled from a distribution, for example, a normal distribution. Three of the most commonly used few-armed bandit tasks are the Two-armed bandit^2^, the Restless bandit^3^, and the Horizon task^4^. The Two-armed bandit and the Restless bandit motivate continued exploratory behavior because the mean rewards of their arms evolve according to a Gaussian random walk. While the mean rewards of all arms in the Restless bandit change on every trial, this is not the case in the the Two-armed bandit. Here, each arm may have drifting mean rewards or not (fully crossed) and participants play several rounds of ten trials. The Restless bandit typically uses four arms over only one round of 200 trials. The Horizon task, a modified Two-armed bandit, addresses the confound between an agent’s estimate of an arm’s value and its associated uncertainty, which typically become correlated over trials due to preferential sampling of the higher-value arm. Through an initial phase of four forced choices, value and uncertainty are perfectly de-correlated on the first free choice across all rounds, providing a statistically cleaner measure of how individuals integrate information about value and uncertainty in their decision-making. The Horizon task further motivates exploratory behavior by manipulating whether participants will be able to use the information gained through exploration. Specifically, after those four initial fixed choices, participants are able to make either one (short Horizon) or six (long Horizon) free choices, making exploration beneficial in the latter but not the former scenario.

In addition to these differences in how the tasks motivate exploration behavior, there are also subtle differences in how exploration strategies are defined in the computational models of these tasks: In the Horizon task, the measurement of exploration is based on a difference score between the two horizon conditions. In particular, directed exploration is defined by the difference between choosing the more informative arm in the long versus the short Horizon, i.e., the degree to which participants seek out information when it can be used later in that round compared to when it cannot be. Value-guided exploration is defined by the difference between the weight relating the expected value difference between the two arms to choice probability in the long versus the short Horizon, i.e., the degree to which participants decide against the arm with the higher expected value. In the Two-armed bandit and in the Restless bandit, directed exploration and value-guided exploration are defined without calculating a difference score. That is, they are merely defined by the degree to which participants seek out uncertain options and by the weight with which they relate the expected value difference to choice probability, respectively. Typically, a different form of exploration, random exploration, is also calculated in the Two-armed bandit. Random exploration is defined by the degree to which participants’ behavior gets less deterministic as overall uncertainty increases, a strategy sometimes referred to as Thompson sampling^2^. In particular, when two options have the same average value difference but different uncertainties, value-guided exploration ignores uncertainty and chooses based only on expected value. Random exploration, however, prefers options with higher uncertainty. As the model becomes more certain about option values, it explores less.

Initially, these three bandit tasks have been used to assess exploration strategies on the population level (see^2,5,6^ for the Two-armed bandit,^3,7–11^ for the Restless bandit and^4,12^ for the Horizon task). Across all three tasks, one main conclusion is that humans use a mixture of value-guided and uncertainty-guided exploration strategies. In other words, individuals tend to explore arms despite lower expected rewards (i.e., value) and arms about which they possess less information (i.e., uncertainty).

Recent work has used few-armed bandit tasks as measurement instruments, i.e. to look at individual differences of exploratory behavior. For example, Fan et al.^13^ showed that people who describe themselves as anxious make less use of uncertainty in their decisions and tend to respond more deterministically. These results and similar ones^14–18^ provide the groundwork for understanding cognition in people with psychiatric conditions, and for providing instruments to improve these conditions therapeutically. However, we only know very little about the psychometric properties of computational measures of cognitive constructs in general (see Karvelis et al.^19^ for an overview and concrete methodological recommendations) and about exploration strategies as inferred using computational model parameters in particular. Specifically, it is yet to be established whether (a) individual differences in model-based estimates of exploration strategies are stable over time (i.e., test–retest reliability), which is a prerequisite for their use as measurement tools, (b) the same exploration strategy is correlated across tasks (i.e., convergent validity), which is a prerequisite for considering the strategy to be a general phenomenon, rather than a task-specific artifact, (c) the strategies are sufficiently distinct from a general, performance-related construct (i.e., divergent validity with respect to working memory), and (d) the strategies are predictive of self-report measures of exploration (i.e., criterion validity).

While some studies showed mediocre to acceptable test–retest reliabilities of model parameters (Four-armed Restless bandit with discrete rewards^20^, probabilistic reversal learning task^21^, Two-armed bandit^22^, predictive inference task^23,24^), to the best of our knowledge, so far no study has evaluated the convergent, external, and criterion validity of model-based exploration strategies. This is problematic, because a lack of reliability and convergent validity can lead to spurious correlations between variables and inconsistent research results such as the inconsistent findings on the relationship between exploration and anxiety^13,15,17,18,25^. It is currently unclear whether the reliability of these measures is sufficient, and whether they indeed measure a general exploration construct, which would allow us to use them in individual difference research.

In general, correlating model parameters derived from a single task, for example with psychometrically developed scales, is problematic for several reasons. First, according to classical test theory, any measure contains error variance. Second, the resulting construct variance can be contaminated by task-specific variance^26^. Third, parameter trade-offs, induced during model fitting^27^, may lead to systematic bias in the estimated model parameters. Individual differences in these parameters can then not be interpreted to reflect variability in the construct in question. In the past, using a broad range of tasks, representing a cognitive construct through a confirmatory factor analysis has been successfully applied to extract general factors of working-memory capacity^26^ and components of reaction-time distributions^28^, and to circumvent the three just mentioned problems. In particular, the confirmatory approach allows us (a) to remove correlations between parameters within a single task induced during the model fitting by modeling correlated residuals, which is, for example, not possible with principal component analysis (i.e., PCA), and (b) to extract generalizable aspects of exploration strategies by modeling the correlations between the same strategy in several tasks, and therefore effectively ignoring task-specific artifacts (e.g., task strategies).

The present work therefore aims to systematically examine the recoverability, test–retest reliability, convergent, divergent and external validity of three exploration tasks. To gauge parameter recoverability, we used separate parameter recovery analyses^29^ for each model and task. We measured test–retest reliability of task measures and model parameters using the intraclass correlation coefficient^30^, and examined the validity of the exploration strategies using structural equation modeling^31^. Additionally, we present specific recommendations of how to improve the measurement of exploration strategies.

## Results

**Fig. 1:**
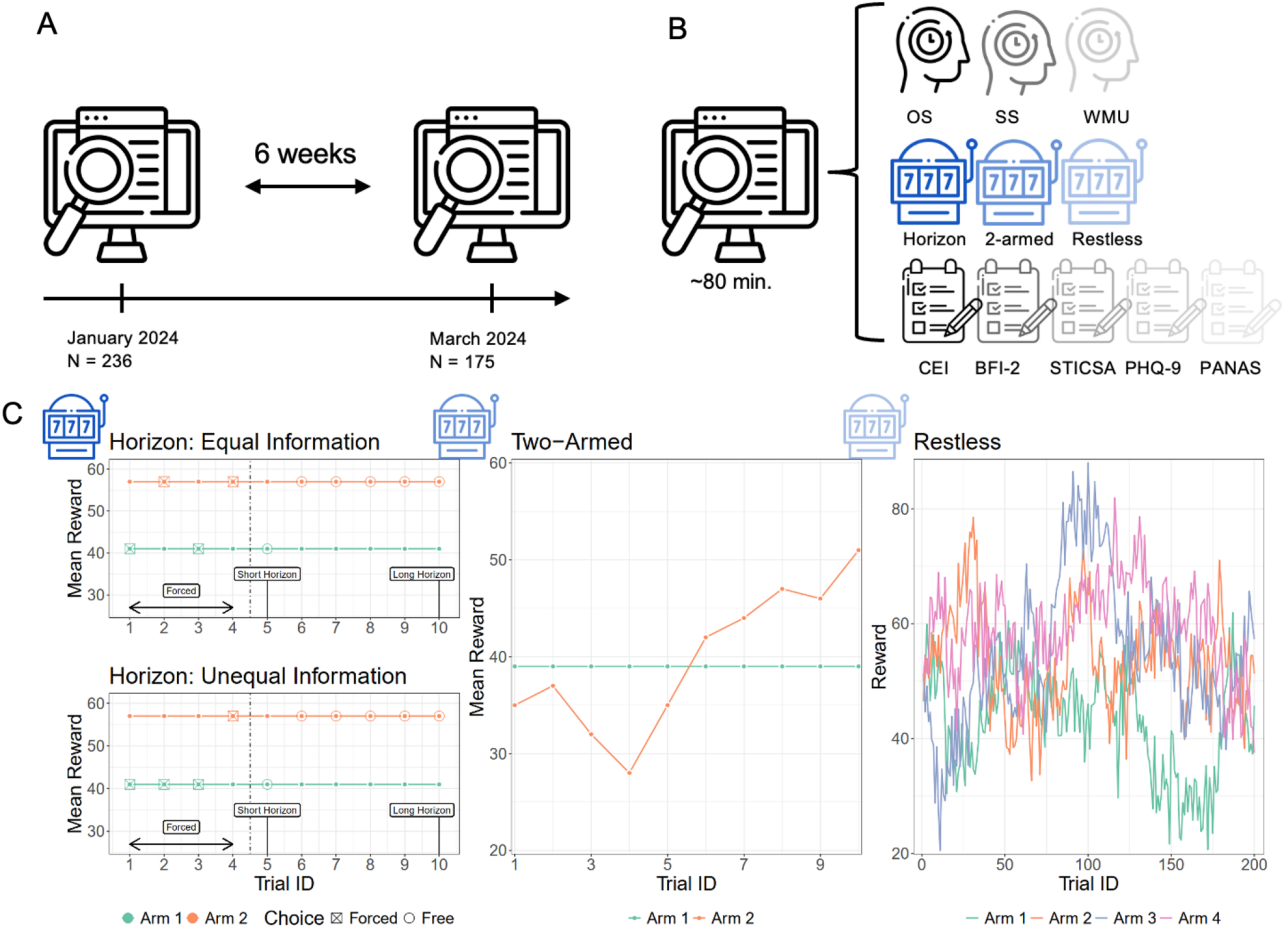
Study overview. (**A**) Our study consisted of two study sessions 6 weeks apart. In the first session, 236 participants passed the inclusion checks and were re-invited for the second session. Out of these 236 participants, 175 participated in the second session and passed the inclusion checks again. (**B**) Each study session lasted for around 80 min and consisted of three working memory tasks, three bandit tasks and five questionnaires. (**C**) Reward structures of the three bandit tasks. In the Horizon task, the two mean rewards were stable within each block. In the Two-armed bandit, either one or both or none of the arms had drifting mean rewards throughout a round. In the Restless bandit, all arms had mean rewards that were continuously drifting throughout the 200 trials. See Methods for details.

### General approach

In each of two sessions separated by 6 weeks, participants completed a series of three working memory paradigms, three few-armed bandit paradigms, and five questionnaires (see Fig. 1). This allowed us to assess the test–retest reliability of all used measures. We used three canonical few-armed bandit paradigms to test whether it is possible to extract generalizable aspects of the proposed exploration strategies (i.e., convergent validity). In particular, we used the Horizon task introduced by Wilson et al.^4^, the Two-armed bandit introduced by Gershman^2^, and the Restless bandit introduced by Daw et al.^3^. In all three bandit paradigms, participants were required to collect as many rewards as possible in a limited number of trials, creating an exploration-exploitation dilemma.

We used three working-memory paradigms to assess whether exploration differs from a general, performance-based ability (i.e., divergent validity). The three paradigms consisted of two complex-span tasks (an operation span task, OS, and a symmetry span task, SS)^32^ taken from the short working-memory capacity battery by Oswald et al.^33^ and a working-memory updating task (modeled after von Bastian et al.^34^). The questionnaires included two scales to assess the external validity of exploration, i.e. the exploration subscale from the Curiosity and Exploration Inventory (CEI)^35^ and the Openness subscale from the short form of the Big Five Inventory-2 (BFI-2)^36^, as well as three scales to assess the criterion validity of the exploration strategies in predicting anxiety and depression, i.e. the State Trait Inventory of Cognitive and Somatic Anxiety (STICSA)^37^, the Patient Health Questionnaire Mood Subscale (PHQ-9)^38^, and the Positive Affect Negative Affect Scale (PANAS)^39^.

To estimate the extent to which participants’ choices were guided by different exploration strategies, we fit those computational models to the choices in the three bandit paradigms based on what has been previously proposed in the literature^2,4,7^. Although the implementation differs slightly across models, they all assume that people sequentially learn the expected values of the arms of the bandit and their uncertainties, and subsequently integrate this information to make a choice. The models in the Horizon task and in the Restless bandit propose that it is only the learned values (value-guided exploration) and their associated uncertainties (directed exploration), which guide people’s choices. The model in the Two-armed bandit additionally assumes that the relation between the learned values and the total uncertainty across both arms (random exploration or Thompson sampling) also affects choices. Despite these conceptual similarities, the models differ slightly in how value-guided and directed exploration are implemented: In the Two-armed bandit, value-guided exploration and directed exploration are implemented as the regression weights of the difference in expected rewards and expected uncertainty, respectively. In the Horizon task, value-guided and directed exploration are implemented as the difference in these regression weights between the long and the short Horizon condition. In the Restless bandit, value-guided and directed exploration are implemented as the inverse temperature and uncertainty bonus in an upper-confidence bound policy, respectively. We implemented all three models as hierarchical Bayesian models (following recommendations by Karvelis et al.^19^), which made parameter estimation more robust, improved out-of-sample prediction and the parameters’ reliabilities, and did not change anything about the individual estimates and group-level patterns (see Tables S3 and S4 and Figures S1 and S2 in the SI). Note that our main analyses and hypotheses as well as the exclusion criteria were preregistered with the Open Science Framework (https://osf.io/cavj3). All task and analysis code as well as all raw data is available under https://osf.io/ra7su/.

### Recoverability

**Fig. 2:**
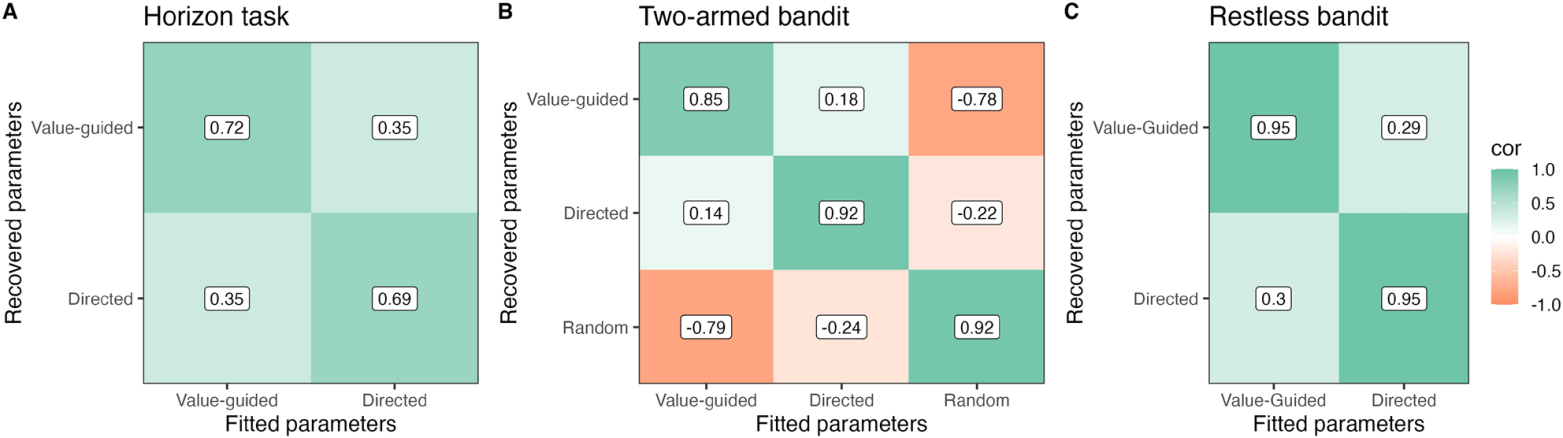
Parameter recovery using the default models for each task. Numbers indicate the correlation between fitted and recovered parameter estimates when using the data from the first session.

We tested the parameter recovery for the computational models in all three bandit tasks by fitting the observed choices from the first session with the respective model, simulating data from the fitted parameters, re-fitting that data and estimating the correlation between the fitted and recovered parameters. All parameters recovered reasonably well (see Fig. 2). Recovery in the Horizon task was notably worse than in the other tasks. We additionally observed a strong off-diagonal correlation in the Two-armed bandit (Fig. 2B) between value-guided exploration and random exploration ($-0.79$). This points towards redundancy in the parameters and multicollinerarity in the predictors. This in turn can reduce the interpretability of the parameter estimates.

### Replicability

**Fig. 3:**
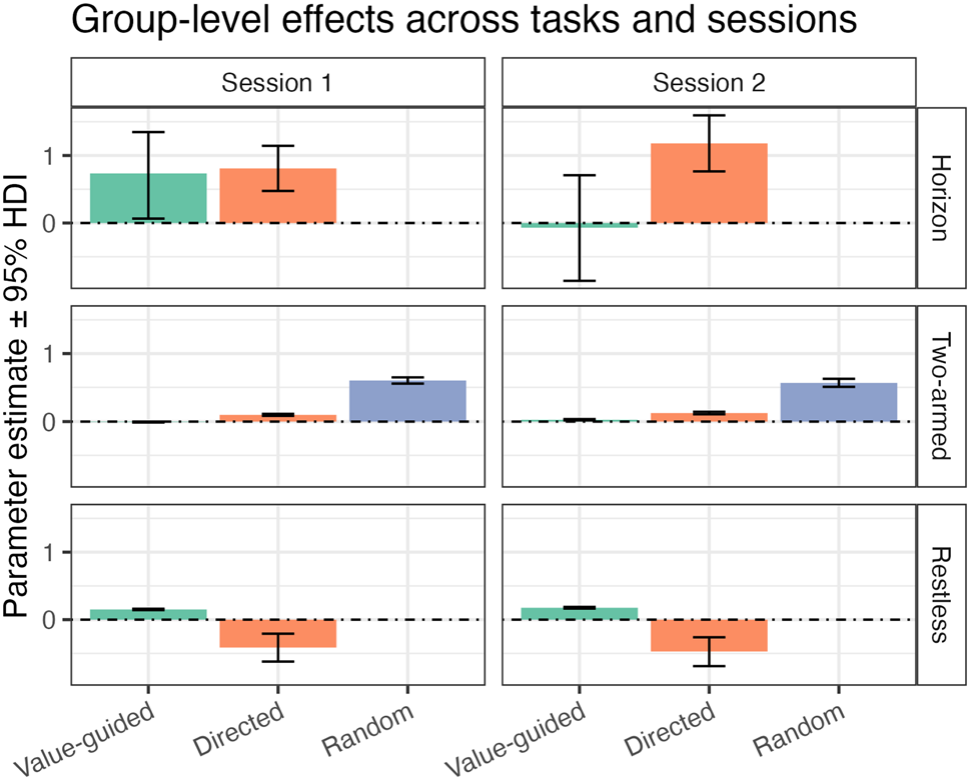
Replicability of the group-level effects. The height of the bars indicates the mean of the group-level posterior distribution for that parameter at that time point. The error bars indicate the 95% Highest Density Interval of the group-level posterior distribution.

We then tested whether the group-level exploration patterns were replicable across time points (see Fig. 3). For the Horizon task, the group-level pattern changed qualitatively, with value-guided exploration disappearing completely in the second session. This was due to the fact that participants used value-guided exploration in the long and the short horizon to the same degree in Session 2, but not in Session 1. Notably, because of the difference calculation of the strategies, variance in the group-level posterior distributions was inflated (see top panel of Fig. 3  ; see also: ^40–42^). For the Two-armed bandit and the Restless bandit, the group-level patterns replicated well across time points. Note however, that for the Two-armed bandit there was virtually no signature of value-guided exploration as all variance was taken up by the highly correlated random exploration parameter. Also note that for the Restless bandit, the group-level parameter for directed exploration was negative, indicating that participants actively avoided uncertain options instead of exploring them. The mean as well as the upper and lower thresholds of the 95% highest density interval of the group-level posterior distributions are displayed in Table 1.

**Table 1:** Means as well as the upper and lower thresholds of the 95% highest density interval of the group-level posterior distributions (values rounded to two decimals).

Task	Session	Strategy	Group-Effect	95% CI Lower	95% CI Upper
Horizon	Session 1	Value-guided	0.74	0.07	1.35
Horizon	Session 1	Directed	0.81	0.48	1.14
Horizon	Session 2	Value-guided	− 0.07	− 0.86	0.71
Horizon	Session 2	Directed	1.18	0.76	1.59
Two-armed	Session 1	Value-guided	− 0.01	− 0.01	0.00
Two-armed	Session 1	Directed	0.10	0.08	0.12
Two-armed	Session 1	Random	0.60	0.56	0.65
Two-armed	Session 2	Value-guided	0.03	0.02	0.04
Two-armed	Session 2	Directed	0.13	0.11	0.15
Two-armed	Session 2	Random	0.57	0.51	0.63
Restless	Session 1	Directed	− 0.41	− 0.62	− 0.21
Restless	Session 1	Value-guided	0.15	0.14	0.16
Restless	Session 2	Directed	− 0.47	− 0.69	− 0.26
Restless	Session 2	Value-guided	0.18	0.17	0.19

### Reliability

**Fig. 4:**
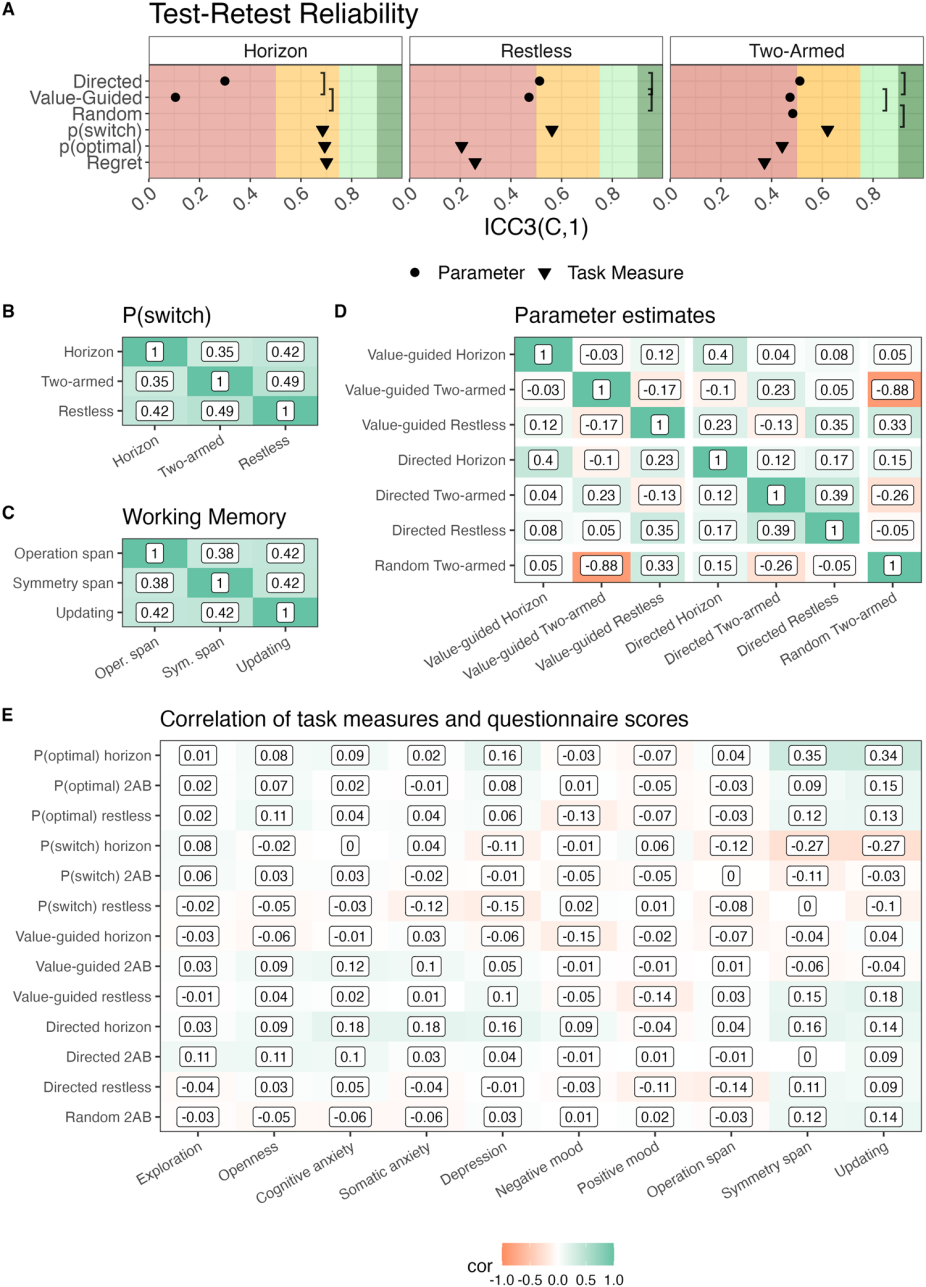
(**A**) Test–retest reliability of model parameters and task measures. The red, orange, light green and dark green background indicates bad, acceptable, good and very good reliability, respectively. Circles indicate the reliabilities of model-based parameters. Triangles indicate the reliabilities of model-free task measures. Leftwards facing square brackets represent the recoverability of the parameters. (**B**) Convergent validity of the trial-by-trial switch probability. Correlations between participants’ average proportion of trials where they selected a different option than on the previous trial across tasks. (**C**) Convergent validity of the recall accuracy in the working memory tasks. (**D**) Convergent validity of the model parameters in the bandit tasks. Correlations between subject-level model parameters across tasks. (**E**) External validity of task measures and model parameters. Correlations of subject-level task measures as well as model parameters with all questionnaire scores and performance in all three working memory tasks. Note: Exploration refers to the CEI, Openness to the BIG5 Openness subscale, Cognitive and Somatic Anxiety to the respective STICSA subscales, Depression to the PHQ-9, and Negative and Positive mood to the respective PANAS subscales.

Next, we evaluated whether the model parameters were consistent over the 6-week period. Therefore, we calculated the Intraclass Correlation coefficient (ICC) between the model parameters estimated at the first and at the second session. Specifically, we focused on the consistency definition of the ICC^30^, because we were mostly interested in the relative amount of exploration irrespective of practice effects. That is, although practice effects may exist in few-armed bandit tasks, our focus was on whether participants who explore more in the first session still explore more in the second session. For the sake of completeness, we also report the results using the absolute agreement definition. In line with previous work on the Two-armed bandit^22^, the model parameters demonstrated mediocre reliabilities at best, with some parameters having a poor reliability^43^. In particular, the ICC3(C,1) of value-guided and directed exploration were 0.105 and 0.300, respectively, in the Horizon task; 0.471 and 0.513, respectively, in the Restless bandit; and 0.473 and 0.511, respectively, in the Two-armed bandit. The results looked similarly when considering the ICC3(A,1), which also considers the average difference in a measure between sessions as a critical source of variability (e.g., practice effects, see Table S5 in the SI). Two exceptions were value-guided exploration and p(optimal) in the Two-armed bandit task. The drop from the ICC3(C,1) to the ICC3(A,1) may reflect the fact that participants generally responded more deterministically in the second session than in the first session. The shared variance between measurement time points ($R^2$) was less than 0.36 for all parameters, and for most even less than 0.27. The low reliability observed in the Horizon task may be partially attributed to the fact that exploration is defined by the difference between two experimental conditions, and difference scores are notoriously known for providing low reliabilities^40–42^. Besides the model parameters, we also analyzed a few model-free task-based measures. The task-based measures, including performance and switch probability, exhibited moderate to good reliability (ICC3(C,1) = 0.21 - 0.70). Remarkably, the switch probability (i.e. the proportion of trials on which participants selected a different arm than on the previous trial) was consistently more reliable than any of the model-based parameters (ICC3(C,1) = 0.56–0.69; see Fig. 4A). We also tested whether there were any practice effects on the proportion of optimal choices in the bandit tasks, and found very small but statistically significant practice effects for the Horizon task and the Two-armed bandit but not the Restless bandit ($t(174) = 2.63, p =.009$, mean diff. $= 0.01$; $t(174) = 6.46, p <.001$, mean diff. $= 0.02$; $t(174) = 1.96, p =.051$, mean diff. $= 0.02$, respectively).

In contrast to the measures of the bandit tasks, retest reliability was good (i.e., ICC3(C,1) > 0.75) for the questionnaire scales and for the working memory measures. Two exceptions were self-reported exploration tendencies (ICC3(C,1) = 0.736) and working memory updating (ICC3(C,1) = 0.723), which, however, still had moderate reliabilities (see Table S7 for all ICC3(C,1) and ICC3(A,1) values of questionnaire and working memory scores). We expected these results since the questionnaire scales have been validated before^35–39^, and several studies have reported good reliability of the used measures in our working memory tasks (e.g.,^33,34,44^).

### Convergent validity

The then tested whether the model parameters of the same exploration strategy correlated across tasks. The parameters showed poor convergent validity (Fig. 4D). Specifically, the value-guided exploration parameters showed very low correlations across tasks with some correlations even being negative (r = $-0.17$ between Restless bandit and Two-armed bandit). For the directed exploration parameters, the correlation between parameters from the Two-armed bandit and the Restless bandit was 0.39. Parameters from the Horizon task were only weakly correlated with the parameters from the other two tasks (0.12 and 0.17). This poor convergent validity in the model parameters can be explained by two factors: Firstly, in the Two-armed bandit task, the model parameters for value-guided and random exploration are strongly negatively correlated and traded off with each other. Secondly, in the Horizon task, the parameters are calculated as the difference between the long and the short horizon, making them conceptually different from the other two tasks. In addition to model parameters, we also tested the convergent validity of a model-free approximation of exploration, namely the switch probability (P(switch), Fig. 4B). This measure of exploration seemed reasonably consistent across tasks with correlations between 0.36 and 0.49. These correlations are also comparable in strength to the correlations between the three different working memory tasks (Fig. 4C).

### External and criterion validity

We only observed relatively small correlations between the model parameters of exploration and questionnaire-based measures of exploration (strongest correlation was .18 between directed exploration in the horizon task and the questionnaire measures of cognitive and somatic anxiety; see Fig. 4E). However, both task-based measures and parameter estimates showed small to moderate correlations with memory capacity (highest r = 0.35). This suggests that measures from the bandit paradigms are affected by individual differences in cognitive capacity (see Collins & Frank^45^ and Collins et al.^46^) .

### Improving the measurement of exploration

The low replicability of the model parameters in the Horizon task, the overall mediocre reliabilities of the exploration strategies in all tasks, and the lack of convergent validity motivated us to perform three changes to the measurement of exploration (see Methods section). Importantly, from here on out, the labels of directed and value-guided exploration in the Horizon task refer to only the estimates of these strategies in the long Horizon condition and not the difference in estimates between the long and the short Horizon.

**Fig. 5:**
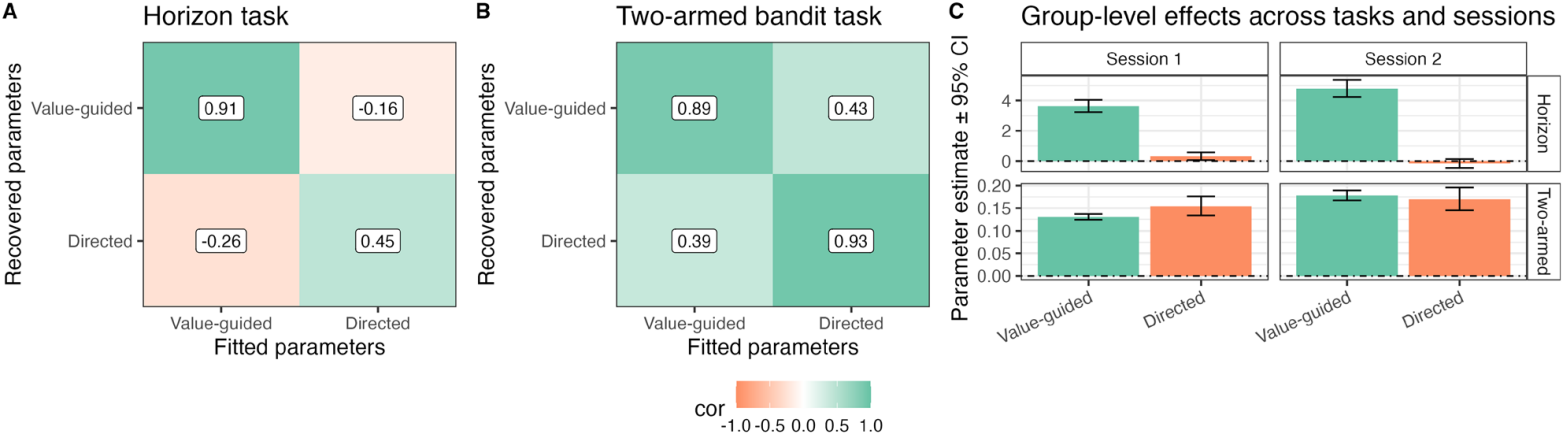
(**A-B**) Parameter recovery following the improvements to the computational modeling, again using data from the first session. Numbers indicate the correlations between fitted and recovered subject-level parameters. (**C**) Replicability of group-level effects from one session to the next following the improvements in the computational modeling. Note that the y axis is on a different scale for the different tasks.

**Fig. 6:**
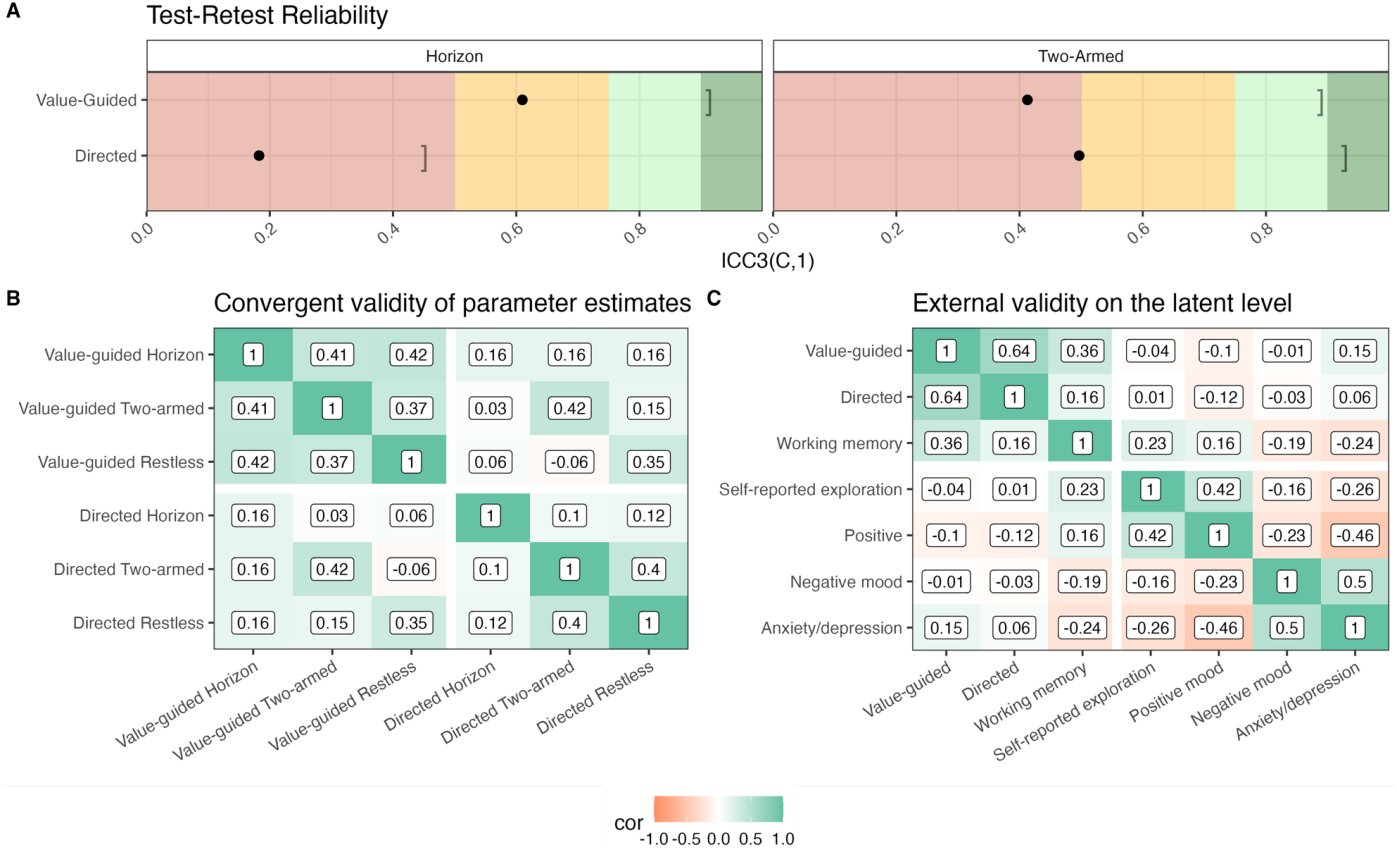
(**A**) Test–retest reliability of model parameters from the Horizon task and the Two-armed bandit following our improvements to the modeling process. Red, orange, light green and dark green backgrounds indicate poor, acceptable, good and very good reliability, respectively. We again calculated the reliabilities in the form the respective intra-class correlation coefficient ICC3(C,1)^30^ as a measure of consistency^52^ (**B**) Convergent validity of the model parameters following improvements on the model fitting process. (**C**) Correlations of latent factors from bandit tasks and questionnaires.

These changes resulted in three desirable effects for the measurement of exploration (see Fig. 5). First, they improved parameter recovery in the Two-armed bandit by reducing the off-diagonal correlations to an acceptable level. Parameter recovery for the Horizon task, however, was on average unchanged. Second, the group-level exploration pattern in the Horizon task was qualitatively replicable across sessions. It is visible in Fig. 5 and Table 2 though, that participants barely used directed exploration on average. A similar pattern was visible on trial 5 of the Two-armed bandit when analyzing choices for each trial separately (see Figs. S3 and S4 in the SI; Note that we validated the fit of the model on a trial-by-trial level and ensured that recoverability was maintained (see Fig. S5)). Hence, the increase in directed exploration from the short to the long horizon, as reported before, reflects rather an avoidance of uncertainty in the short horizon that is attenuated in the long Horizon as opposed to active uncertainty-seeking in the long Horizon. Third, while the convergent validity of directed exploration stayed about the same, the one of value-guided exploration improved (see Fig. 6B). Finally, the changes, on average, did not affect the reliabilities of the model parameters, as their positive and negative effects were roughly balanced. That is, the reliability of value-guided exploration in the Horizon task slightly increased (0.610), but the one of directed exploration slightly decreased (0.183), and the reliability of both strategies in the Two-armed bandit slightly decreased (0.413 and 0.497 for value-guided and directed exploration, respectively, see Fig. 6A and Table S6 in the SI).

**Table 2:** Group effects and confidence intervals for different strategies across sessions and tasks after improvements to the modeling (values rounded to two decimals).

Task	Session	Strategy	Group-Effect	95% CI Lower	95% CI Upper
Horizon	Session 1	Value-guided	3.62	3.23	4.04
Horizon	Session 1	Directed	0.32	0.06	0.57
Horizon	Session 2	Value-guided	4.79	4.23	5.37
Horizon	Session 2	Directed	− 0.15	− 0.45	0.13
Two-armed	Session 1	Value-guided	0.13	0.12	0.14
Two-armed	Session 1	Directed	0.15	0.13	0.18
Two-armed	Session 2	Value-guided	0.18	0.17	0.19
Two-armed	Session 2	Directed	0.17	0.15	0.20

Although the correlations between directed exploration across bandits stayed about the same, those for value-guided exploration increased (see panel B in Fig. 6). This allowed us to evaluate *construct validity*. Therefore, we first created separate measurement models for working-memory capacity, value-guided exploration, and directed exploration on the data from session 1 using the lavaan package^47^ in R^48^. They also allowed us to quantify the contribution from each individual model parameter to the construct via the standardized factor loadings, which are shown in Table 3. Even though all three tasks contributed approximately equally to working-memory capacity and value-guided exploration, it is visible that directed exploration was mostly accounted for by the Restless bandit, to a much smaller degree by the Two-armed bandit, and only negligibly by the Horizon task. We then compared two structural models to test whether value-guided and directed exploration differ on the latent level. The first model included separate latent factors of value-guided exploration and directed exploration. The second model included only one general factor of exploration, with all six model parameters loading on this general factor. Because the first model converged with a negative variance on the session 1 data, but not on the session 2 data, we used the latter for the model comparison. The fit indices of both models are shown in Table S1 in the SI. We approximated the Bayes factor with the method described in^49^. The evidence was clearly in favor of the two-factor model, with a Bayes factor > 1000, supporting the claim that value-guided exploration and directed exploration are separate strategies. Only the latent factor for value-guided exploration converged in both sessions, allowing us to assess the temporal stability of this factor, which was very high (r = 0.78). The model that includes both directed and value-guided exploration only converged for the data from session 2, not session 1, suggesting that directed exploration might only emerge after sufficient exposure to the task.

**Table 3:** Standardized factor loadings for the three measurement models of working-memory capacity, value-guided exploration, and directed exploration using the data of Session 1.

Model	Indicator	Standardized loading
Working-Memory Capacity	Operation Span	0.625
Working-Memory Capacity	Symmetry Span	0.583
Working-Memory Capacity	Updating	0.647
Value-Guided	Horizon	0.597
Value-Guided	Two-armed	0.535
Value-Guided	Restless	0.696
Directed	Horizon	0.137
Directed	Two-armed	0.444
Directed	Restless	0.891

To evaluate *divergent validity* and *external validity*, we initially created a structural model including the two latent exploration factors and a working-memory capacity factor. We then correlated these factors with each other as well as with the two questionnaire measures of exploration. All correlations between the exploration factors and the questionnaire measures of exploration were small and statistically not significant (all ps > .05). Specifically, directed exploration correlated to 0.15 with the CEI scale and to $-0.01$ with the BIG5 openness subscale. Value-guided exploration correlated to 0.05 with the CEI scale and to $-0.13$ with BIG5 openness subscale. The two latent exploration factors were however highly correlated with each other (r = 0.64, p $<.001$) and with the latent working memory capacity factor (r = 0.16, p = .004 and r = 0.37, p $<.001$ for directed and value-guided exploration, respectively; note that the correlations between the latter three relationships remain significant when using Bonferroni’s correction for multiple comparisons).

To further assess external and criterion validity, we created a structural model including a latent factor for self-reported exploration, two latent factors for positive and negative mood, respectively, and a latent factor for anxiety and depression based on participants’ compound scores for each questionnaire subscale. This choice of factor structure was motivated by the similarity of the theoretical constructs as well as their strong observed correlation (r = 0.57, p $<.001$). The factors for positive and negative mood only consisted of the corresponding PANAS subscale (Note: No other confirmatory model combining these subscales with other scales in a latent factor converged.) Lastly, combining depression, somatic anxiety and cognitive anxiety in one factor has both a strong theoretical basis in the computational psychiatry literature^50,51^ and is motivated by the large correlations between these three measures (all r $> 0.64$, all p $<.001$). The fit indices of that model can be found in Table S2 in the SI.

When investigating the correlations between the task-based and questionnaire-based latent factors, we found all correlations to be very small and again not statistically significant (all ps > .05), underscoring the absence of a link between the self-report measures and task behavior (see Fig. 6C). Interestingly, we observed relatively strong correlations between the latent factor for working memory capacity and the latent factors for self-reported exploration and anxiety/depression (r = 0.23 and $-0.24$, respectively). Note that only the latter remained statistically significant after using Bonferroni’s correction. Together with the positive correlations between working memory capacity and both exploration strategies, these relationships point towards a role of working memory capacity in explaining the link between psychiatric traits and behavior on bandit tasks found in the literature.

### Do these tasks actually promote exploration?

To answer this question, we tested which combination of model parameters yields maximum rewards. We tested this for both reward sets in our Study (i.e., Session 1 and Session 2) and as a comparison for reward sets from the literature (or randomly generated reward sets for the Restless bandit). Across all three tasks and all reward sets, we observed that increased value-guided exploration yielded increased rewards, albeit leveling off eventually. Participants’ levels of value-guided exploration were typically slightly below the optimal levels in our datasets (see Figs. S6–S8 in the SI). Directed exploration strongly increased rewards in all reward sets for the Restless bandit (despite participants avoiding uncertainty, a suboptimal strategy). It was only slightly beneficial in the Two-armed bandit task for our reward sets and not at all for the reward set taken from Fan et al.^13^. It had no bearing on the rewards in the Horizon task, neither in our reward sets, nor in the reward set taken from Zajkowsky et al.^12^. This could potentially reflect the influence of drifting versus stable rewards in promoting exploration behavior.

Because the directed exploration model parameter had no bearing at all on the reward earnings in the Horizon task, we investigated more in detail, whether choosing a less explored option in the first free choice during the long horizon could actually change participants’ estimate about which option is best or whether participants already have enough information after the four fixed choices. We found that, in our two reward sets, selecting the less explored option in the long horizon never changed which option was estimated to be more rewarding. Thus, there was indeed no benefit of exploration. In the reward set by Zajkowsky et al.^12^, selecting the less explored option in the long horizon changed the best option on $12.5\%$ of all rounds. Out of these rounds, this update actually made participants more accurate in $74.7\%$ of cases, meaning that overall participants’ estimate of which option was the better one was only improved on $9.3\%$ of unequal information, long horizon rounds.

Besides the just mentioned exceptions in the Horizon task, we can therefore conclude that the task set we used in general motivates exploratory behavior.

## Discussion

We tested the reliability and validity of model-based exploration strategies in a large-scale individual differences study. In line with recent concerns about the psychometric limitations of computational tasks in psychiatry^19^, we observed mixed results: While we observed good parameter recovery, there were substantial parameter trade-offs and weak signatures of directed exploration. Additionally, test–retest reliability was mediocre at best for all three tasks, and convergent and external validity were poor. To address these issues, we refined the measurements of exploration strategies by removing a highly-correlated strategy in the Two-Armed Bandit and only using data from the long horizon in the Horizon task. Furthermore, we calculated latent factors to eliminate task-specific, error, and model-fitting induced variance.

Following these improvements, test–retest reliability of single model parameters remained mediocre, but we successfully extracted a temporally stable latent trait of value-guided exploration, with a reliability of 0.78. Moreover, we identified a latent factor of directed exploration in the second session, but not in the first session, which may indicate that participants converged upon a strategy after sufficient task exposure. This result, together with the non-uniform distribution of factor loadings, also points towards the difficulty in measuring a stable trait of directed exploration. In particular, these observations highlight challenges in obtaining approximations of directed exploration using parameters from single tasks with single time point measurements and correlating them with other measures (e.g., personality), as is the default approach in the literature. Furthermore, compared to Anvari et al.^53^, who found no converging evidence of exploration tendencies using model-free task measures, our results paint a different picture. How is that possible? One possible reason for the discrepancy is that our selection of tasks was constrained to few-armed bandits, while Anvari et al. also included other paradigms (e.g., optional stopping, decisions from experience). Thus, the identified individual differences on the latent level may only be generalizable to situations actually reflecting an explore-exploit trade off (e.g., restaurant selection), but not to optional stopping scenarios (e.g., job search). Our results are again partially in line with Jach et al.^54^ who also successfully extracted a latent factor of value-guided and directed exploration from a set of nine behavioral tasks. However, while the two strategies represented different ends on the same latent factor in their study, they were highly positively correlated in our data. Possible explanations for this difference could be the different modeling framework and that we systematically excluded task-specific variance from latent exploration strategies using the CFA approach.

Furthermore, that high correlation between directed and value-guided exploration points to potential problems in distinguishing between the two strategies. The strategies were, however, sufficiently distinct for the structural model with separate latent factors to be a better fit to the data than a model with just one exploration factor, providing evidence that the two are indeed distinct exploration strategies. A grain of salt on top of that interpretation in our study, however, is that people rarely actively engaged in directed exploration. In fact, the respective model parameters were negative in the Restless bandit, they turned negative after five choices in the Two-armed bandit (see Figs. S3 and S4 in the SI), and they were indistinguishable from zero on the first free choice in the Horizon task. This pattern is more in line with the active avoidance of uncertainty on most trials.

Given the consistently observed negative sign of directed exploration parameters, a critical question arises: Can directed exploration truly be estimated in these types of bandit tasks? Or do these tasks even motivate people to explore? We tested what combinations of model parameters would give the best rewards on our reward sets, as well as on comparison reward sets (see Figures S6 through S8 in the SI). We found that in the Restless bandit and in the Two-armed bandit, a certain degree of directed exploration increases cumulative rewards, likely due to the volatility of the rewards. This was not the case for the Horizon task, where rewards were stable. These patterns were the same when testing a random reward set or reward sets taken from the literature^12,13^. This thus suggests that, given the currently used task settings, only the tasks with changing rewards create a continued need for exploration as otherwise the ideal arm is apparent after very few choices. Additionally, we observed that in the Two-armed bandit, only our reward sets and not the reward set from the literature^13^ promoted exploration. During piloting of our study, we saw that it was important that the reward differences between arms were small enough to promote exploration but not too small that performance becomes indistinguishable from chance. This may partially explain why there are differences in what behavior is optimal between the different reward sets. Lastly, we tested whether participants’ estimates of which arm provides higher average rewards were actually changed by exploring the less explored option in the long horizon condition of the Horizon task. We found that in our reward sets, this was never the case, while it was the case on 12.5% of the rounds in the reward set from the literature^12^ (albeit only providing a more accurate estimate of which arm is best on less than 10% of rounds). This difference between our reward set and the reward set from the literature is likely due to the higher noise level in the latter. Thus, the amount of noise added to mean rewards could be another relevant factor in designing tasks that promote exploration.

In sum, tasks with drifting rewards, smaller reward differences and larger noise levels - while still not being too difficult - promote exploration. Another potential way forward is to use these tasks but limit the number of trials per round (e.g., stopping after five choices). This approach might help to isolate the effects of directed exploration. Another promising direction could be to develop tasks that de-correlate exploration from exploitation, similar as the observe-or-bet task^55,56^. This type of task design could provide clearer insights into the distinct mechanisms of exploration. Additionally, more complex or gamified tasks might offer a better framework for studying exploration strategies^57,58^.

One limitation to our study is that we made several non-negligible modifications to the Horizon task when compared to its original implementation^4^. First, we reduced the total number of rounds from the original 160 rounds to 80 rounds. We verified through a priori power analyses that this reduced trial number would be sufficient to detect expected effects. We additionally performed recovery analyses using simulated data of a varying number of rounds to ensure that in principle, reasonable parameter recovery could be expected from 80 rounds. The results from these simulations as well as a body of literature using versions of the Horizon task with reduced numbers of rounds^15,16,59^ encouraged us that 80 rounds should be a sufficient task length for the Horizon task. Second, in our implementation of the Horizon task participants were not provided with a history of their previous rewards, including the rewards received during the fixed choices. While this implementation has been successfully used before^60^, it adds a working memory load compared to the classic implementation, limiting comparability to previous studies. The choice to omit a reward history was motivated by considerations of comparability across tasks. Third, we altered the computational modeling of the Horizon task slightly to include two separate regression weights for value difference and information difference in a Bayesian mixed-effects regression as opposed to the general choice temperature applied to all predictors in the original implementation^4^. This choice was again motivated by the comparability between bandit tasks and follows a modeling approach suggested by Wilson et al.^61^. Simulations showed the original model parameters to be highly correlated with the model parameters in our implementation (see Table S8 in the SI). While all these alterations were performed to improve the comparability between bandit tasks while maintaining a conceptual, even if not direct, comparability to previous studies of the Horizon task, it is possible that the inferior recoverability and reliability of the Horizon task could be driven by these limitations.

Notably, value-guided exploration and self-reported exploration were highly correlated with working memory capacity. This is in line with the observation that participants whose working memory was taxed with a secondary task (i.e., lower capacity), showed decreased value-guided exploration on the horizon task^60^. This strong correlation with a general, performance-based cognitive construct, points towards general problems in the measurement of individual differences using behavioral tasks (for similar issues in the measurement of individual differences in category learning, see Lewandowsky^62^ and Lewandowsky^63^). How much meaningful variability between people can we extract from behavioral cognitive paradigms, and few-armed bandits in particular? The extent to which we can expect to measure meaningful variance in cognitive constructs depends on clear theoretical definitions^64^, and on how we derive measurement instruments in accordance with this definition^65^. It also depends on the development and re-use of standardized tasks^66^. In this study, we hope to provide a starting point in this endeavor through our reward sets, which were optimized to detect individual differences in behavior and fixed across participants, and our improved model versions. Notably, the latent factor for anxiety and depression was also highly correlated with working memory capacity, posing the question of whether common findings on the link between anxiety and depression and exploration behavior^13,14,17,18^ might be, at least partially, driven by individual differences in working memory capacity.

Moreover, none of the factors derived from the bandit tasks were associated with the questionnaires or questionnaire-derived factors. This lack of association suggests that the behavioral and questionnaire-based constructs measure distinct aspects of individual differences. This observation adds to a growing body of research showing that behavioral tasks and questionnaires capture qualitatively different aspects of behavior^67^ (self-control^68,69^, thought control^70^ and risk^71^). It could be, as has been pointed out^67^, that the task-based exploration strategies represent an upper bound on exploration, while the questionnaire-based exploration strategies reflect average exploration, affected by motivation and other factors. Another option is, similar to the domain of risk^72^, that exploration is domain-specific. Then, new domain-specific instruments would have to be developed to predict domain-specific exploration behavior.

Finally, it is worth considering whether humans necessarily use these principled exploration algorithms. The history of these algorithms stems from artificial agents^73,74^ that require exploration strategies to function effectively, for example, to avoid getting stuck in a local minimum. However, just because artificial agents need these kinds of strategies does not mean that humans need to use these exact strategies. Instead, they could rely on more heuristic approaches rather than explicitly computing their uncertainty about each option^9,25,75^. In fact, the difference between the rewards earned using the optimal strategy and a heuristic strategy is usually no more than a few cents. It therefore remains an open question whether participants in these experimental studies are simply not incentivized enough to seek maximum rewards^76^, or whether these complex strategies are simply not a good representation of how people make (real-world) choices.

In conclusion, the lack of robust external and criterion validity observed in our study raises important questions regarding the interpretability and practical relevance of model-based exploration measures. While we were able to extract stable latent constructs–particularly for value-guided exploration–these constructs were not predictive of self-reported exploration tendencies, mood, or psychiatric symptoms. This disconnect suggests that, even with improved modeling and latent variable techniques, the external validity of these paradigms remains uncertain. If such tasks fail to capture variance that generalizes to real-world behaviors or clinically relevant constructs, their value as tools for individual differences research may be limited. At a minimum, these findings call for a reevaluation of the assumptions underlying current task designs and their intended applications. Future work may benefit from developing tasks that more strongly decouple exploration from performance demands, possibly incorporating richer, ecologically grounded scenarios, or explicitly modeling domain-specific forms of exploration.

## Conclusion

We measured value-guided and directed exploration using three bandit tasks. Our findings suggest that using all three tasks provides a comprehensive assessment of a stable trait of value-guided exploration. If constraints limit the use of all tasks, the Restless bandit task is recommended due to its highest factor loadings. Despite our ability to identify a factor of directed exploration, the negative parameter values indicate that these bandit tasks may not be well-suited for studying directed exploration. The data suggest that participants tend to converge quickly on a preferred option, exhibiting minimal exploration behavior. This rapid convergence highlights a potential limitation in using these tasks to investigate directed exploration comprehensively.

## Methods

### Participants

We invited participants on the Prolific platform (prolific.com) who had completed at least 5 studies prior to participation, had an acceptance rate of at least 95%, were between the ages of 18 and 50, reported English as their primary & first language, and did not report any language-related disorders. Prior to participation, they all gave informed consent and were informed about the privacy policy and their payment. Payment consisted of a fixed portion (GBP 9 for each session) and a bonus portion (up to GBP 6 for each session) that rewarded performance on the behavioral tasks but not the questionnaires. The study was performed in accordance with the relevant guidelines and regulations approved by the ethics committee of the University of Tuebingen (project nr. 202/2023B02, study title: Psychopathology and cognitive processes). Experiments were presented to participants using a combination of HTML, javaScript, CSS with custom code, and jsPsych^77^. For the working-memory tasks, we adapted code made available by Luthra & Todd^78^.

In session 1, we collected data from 357 participants. We subsequently excluded participants who failed any of the following inclusion criteria: A full dataset, no use of external aids during the working memory or bandit tasks (as judged by self-report), bandit task performance better than the 95th percentile of chance performance as given by a binomial distribution, fewer comprehension attempts during the bandit task portion than the mean + 2SD (such as to exclude participants who were merely guessing the correct answers instead of reading the instructions), correctly answering both of the two attention check questions in the questionnaire portion and performing better than the 95th percentile of chance performance in the processing portion of the working memory tasks. On the basis of these exclusion criteria, we selected a total of 236 participants, whom we invited back for a second session. We subsequently applied the same criteria to the data we gathered from the second session, resulting in a final sample of 175 participants (84 females, mean age = 34.23, SD = 7.77).

### Behavioral paradigms and questionnaires

#### General procedure

Both sessions followed the same sequence of tasks: participants started with the three working-memory tasks, which were presented in the same order for every participant, followed by the three bandit tasks, followed by the five questionnaires all presented on the same page. We presented the bandit tasks in randomized order across participants but fixed the order across sessions for every individual participant. The reasoning was the following: Order effects can impact the reliability and precision of our results^79^ (for example, if the order effect is reflected in an increase of inattentive responding over time leading to a more noisy distribution in later tasks). Additionally, completely randomizing tasks within a single participant complicates the interpretation of factor loadings across sessions. To mitigate these issues, we randomized the order of bandit tasks across participants but kept the order fixed across sessions for each individual participant.

In the Horizon task and the Two-armed bandit, participants selected a slot machine by pressing S or K on the keyboard, in the Restless bandit, they selected a slot machine with S, D, K, or L. In the working-memory updating task and in the operation span task, participants recalled the memoranda by typing on the keyboard. In the symmetry span task, they recalled the locations by sequentially clicking with the mouse on the respective grid cells; in both processing parts, participants responded with the up (correct) and down arrow keys (incorrect) on the keyboard. The stimulus sets (memoranda and processing items in the working-memory tasks and rewards in the bandit tasks) were pre-sampled and the same for all participants. We decided upon the final set sizes in the working-memory tasks and all reward sets in the bandit tasks in an initial pilot study to avoid ceiling and floor effects.

#### Bandit tasks

*Horizon task.* We adapted the same general procedure as in the original publication^4^ with two modifications: First, the history of rewards was not available to participants, to ensure comparability between the bandit tasks, an implementation that had been successfully applied in the literature^60^. Second, participants played one practice round of the task which was followed by only 80 task rounds, as opposed to the originally proposed 160. This was driven by considerations for the total length of the experiment and participants’ attention span. Prior to data collection, we conducted a power analysis based on publicly available data by Zaller et al.^59^ and found that the group-level effects in that dataset could be recovered 100% of the time when using a total of 80 games. We additionally tested the correlations between original and recovered parameter estimates on the individual level for different numbers of games and found only minor differences between the versions for 80 and 180 games (r = 0.81–0.90 vs r = 0.88–0.94).

In each round, there was a message above the slot machines indicating whether they were playing a long or a short round. During the first four trials, participants had to select the slot machine that was highlighted for them. After these initial four forced choices, the slot machines briefly disappeared from the screen and there was a message reminding the participants whether they were about to make one or six free choices, depending on whether they were in a long or a short round.

When generating the reward set for the Horizon task, we fully crossed Horizon length and available information and made sure that each of the four resulting conditions had the same reward on average. We also ensured that both arms were on average, across the entire task, equally rewarding. Finally, we ensured that each combination of the Horizons and information conditions was equally difficult by assigning the same average reward differences to each combinations. Those differences were 30, 20, 12, 8, and 4, as these were the differences that avoided both ceiling and floor effects during piloting.

*Two-armed bandit.* We followed the procedure outlined by Fan et al.^13^, which adapts the task originally proposed by Gershman^2^. Participants chose freely between two slot machines for 30 rounds, each consisting of 10 choices. The reward conditions varied: sometimes both arms had stable mean rewards, sometimes only one arm was stable, and sometimes both arms had mean rewards that drifted according to a random walk (for details, see Fan et al.^13^). For the drifting arms, we ensured that the average reward differences between the two arms did not exceed 15 over an entire round. Additionally, we biased the means of the generating reward distributions towards an 8-point difference, which proved effective in distinguishing between participants during piloting. Note that arms were not explicitly labeled as constant or drifting, which represents a change from^13^.

*Restless bandit.* In the Restless bandit task^3^, participants could freely choose between four slot machines. They played one round that lasted for 200 choices. The rewards were sampled from four randomly walking mean rewards with standard deviation of 4. The mean rewards were sampled the same as in Daw et al.^3^ according to $\mu _{i,t+1} = \lambda * \mu _{i,t} + (1 - \lambda )*\theta + v_{innov}$, with lambda set to .9836, the decay center set to 50, with the only exception that the innovation variance / diffusion noise was set to the lower value of 7.84 (sd = 2.8). We initially sampled several random walks and then finally selected two random walks such that there was variability in the which arm was the best arm across trials, and we made sure that each arm was never best for an extended period to motivate exploration.

While the changes compared to the source studies in the Horizon task (i.e., no history) and in the two-armed bandit (no labeling of the arms) prohibited a direct replication of the original studies, the current task set represents a conceptual replication^80^ of the source studies. That allowed us to test whether the previous observations are generalizable to slightly different task setups.

#### Working memory tasks

*Operation span and symmetry span.* In both tasks, the presentation of memoranda was interleaved with the presentation of distracting processing problems. Memoranda were presented for 1000 ms, processing problems for maximally 6000 ms, but they were replaced immediately with the next memorandum after participants had responded to a given problem. Participants were instructed to recall the memoranda in order of presentation and to respond to the processing problems as accurately and as quickly as possible. In the operation span task, set size varied from 4 to 8, in the symmetry span task from 3 to 6. Every set size was tested twice in each session. We used a strict scoring scheme for calculating the proportion of correct responses. That is, only memoranda recalled in the serial position, in which they were presented, were scored as correct.

The memoranda in the operation span task were selected from all consonants (with the exception of J and Y), those in the symmetry span task were selected from the possible 16 locations in a 4x4 grid. Processing problems consisted of the validation of equations (additions and subtractions, the result always being a one-digit number) in the operation span task and of the judgment of symmetry of patterns along the vertical axis in an 8x8 grid in the symmetry task. The problems were constructed in the following way: half of the problems were correct, half incorrect. Incorrect equations in the operation span task were created by randomly adding or subtracting 1 or 2 to a correct equation. Asymmetric patterns were created by randomly changing 3 or 4 grid cells in one half of the grid.

*Working-Memory Updating.* In every trial, five digits (from 0 to 9) were presented in five adjacent, differently colored boxes in the middle of the screen for 5000 ms. In each of the following seven updating steps one of the five digits was replaced by a randomly sampled digit from the same set. The digit to be updated was presented for 1250 ms within the respectively colored box. We added an inter-stimulus interval of 250 ms in between presentation of the initial set and the first updating step as well as between the remaining updating steps. Participants were instructed to recall the five final digits in order of presentation. As for the other two working memory tasks, we used strict scoring. There were 20 updating trials in total. We added five trials without updating, in which participants were instructed to recall the initial set immediately to ensure correct encoding of the initial set (see^34^).

#### Questionnaires

We used two different questionnaires to investigate real-world exploration behavior: The Curiosity and Exploration Inventory (CEI)^35^ and the Openness subscale from the Big Five Personality Inventory-2 (BFI-2)^36^. The CEI comprises two subscales, namely exploration and absorption. Here, we only used the former, which consists of four items to be rated on a 7-point Likert scale. The Openness subscale from the BFI-2 comprises six items, which are rated on a 5-point Likert scale.

In addition, we administered two psychiatric questionnaires to gain insight into the levels of anxiety and depression experienced by the participants. With regard to the former, we opted to utilize the trait version of the State Trait Inventory of Cognitive and Somatic Anxiety (STICSA)^37^. It comprises 21 items in total, 10 of which pertain to cognitive anxiety and 11 to somatic anxiety. Participants were invited to indicate their level of agreement with each item on a 4-point scale, ranging from “almost never” to “almost always”. We assessed depressivity using the Patient Health Questionnaire Mood Subscale (PHQ-9)^38^. It consists of nine items, which are rated on a four-point scale ranging from "not at all" to "nearly every day".

Finally, we were interested in exploring whether behavior on the bandit tasks is influenced by transient fluctuations in mood. We therefore assessed positive and negative mood using the Positive Affect Negative Affect Scale (PANAS)^39^. This scale consists of 20 adjectives, 10 describing positive affect and 10 describing negative affect. Participants were invited to rate how much each adjective described the way they were feeling at that moment on a 5-point scale ranging from "very slightly or not at all" to “extremely”.

#### Computational modeling

We used the same computational models as proposed in the source studies. For the Horizon task, we used a model based on the model proposed by Wilson and colleagues^4^ modeling only the first free choice in every round after the four forced choices.

As compared to the other two bandit tasks, learning was not implemented in an incremental fashion but only once for the fifth choice (i.e. the first free choice). The expected value of arm j, $E_j(t=5)$, was calculated as the average of the observed rewards during the forced choices. $I_j(t=5)$ was an indicator variable tracking the accumulated information about each arm j (i.e. how often it has been selected):

1 $$\begin{aligned} E_j(t=5)= \frac{\Sigma _{t = 1}^4 \delta _j(t) * r(t)}{\Sigma _{t=1}^4 \delta _j(t)} \end{aligned}$$

2 $$\begin{aligned} I_j(t=5)= \Sigma _{t=1}^4 \delta _j(t) \end{aligned}$$

where *r*(*t*) is the reward received on trial t and $\delta _j(t)$ = 1 if arm j was chosen on trial t, and 0 otherwise.

In this approach, exploration is defined as the difference in the model parameters between the long horizon and the short horizon conditions. For value-guided exploration, that is reflected in the difference between the parameters relating $E_1(t=5) - E_2(t=5)$ to choice probability. For directed exploration, it is reflected in the difference between the parameters relating $(I_1(t=5) - I_2(t=5))/4$ to choice probability (we divided the difference by 4 to create an indicator variable with levels -0.5, 0, 0.5 to aid with fitting in the logistic regression). For the Two-armed bandit, we used the model proposed by Gershman^2^ (with the sole difference that we used a logistic regression instead of a probit regression for convergence reasons), and for the Restless bandit, we used the Kalman filter combined with the "softmax with exploration bonus" choice rule introduced by Speekenbrink and Konstantinidis^7^.

Accordingly, in both of those tasks, we used a Bayesian updating rule, i.e., the Kalman filter^81,82^, to learn the expected value $E_j(t)$ and the variance $V_j(t)$ of the average reward on a given arm j on trial t

3 $$\begin{aligned} E_j(t)&= E_j(t-1) + \delta _j(t) * K_j(t) * [r(t) - E_j(t-1)]\end{aligned}$$

4 $$\begin{aligned} V_j(t)&= [1 - \delta _j(t) * K_j(t)] * [V_j(t-1) + \sigma _{innov}^2] \end{aligned}$$

where *r*(*t*) is the reward received on trial t, $\sigma _{innov}^2$ is the innovation variance, and $\delta _j(t)$ = 1 if arm j was chosen on trial t, and 0 otherwise. The learning rate $K_j(t)$, i.e., the Kalman gain, is calculated as follows:

5 $$\begin{aligned} K_j(t) = \frac{V_j(t-1) + \sigma _{innov}^2}{V_j(t-1) + \sigma _{innov}^2 + \sigma _{noise}^2} \end{aligned}$$

Note that $\sigma _{innov}$, $\sigma _{noise}$, $E_j(0)$, and $V_j(0)$ were set to the generating values and not fitted (see Danwitz et al.^83^, Gershman^2^, and Speekenbrink^84^). Then, considering the diffusion process, the priors $E_j(t+1)$ and $V_j(t+1)$ are updated in the following way before making the next decision:

6 $$\begin{aligned} E_j(t+1) = \lambda * E_j(t) + (1 - \lambda )*C_{decay} \end{aligned}$$

with $\lambda$ and $C_{decay}$ referring to the decay rate and the decay center of the diffusion process, respectively. We updated the priors according to the diffusion process only for the restless bandit (see Daw et al.^3^), but not for the Two-armed bandit (see Gershman^2^).

The choice model for the restless bandit was an upper-confidence bound policy with a free inverse temperature parameter $\tau$ and a free directed exploration parameter $\beta$:

7 $$\begin{aligned} P(C(t) = j) = \frac{exp(\tau * E_j(t) + \beta * sqrt(V_j(t) + \sigma _{innov}^2))}{\sum _{k = 1}^4 exp(\tau * E_k(t) + \beta * sqrt(V_k(t) + \sigma _{innov}^2))} \end{aligned}$$

The choice models in the Horizon task and in the Two-armed bandit were implemented via a logistic regression:

8 $$\begin{aligned} P(C(t) = j) = \frac{1}{1 + exp(-(\beta _0 + \varvec{\beta } * \varvec{x}))} \end{aligned}$$

with $\varvec{x}$ containing the independent variables $E_1(t) - E_2(t)$, $\sqrt{V_1(t)} - \sqrt{V_2(t)}$ (or $I_1(t) - I_2(t)$ in the case of the Horizon task), and $\varvec{\beta }$ containing the respective parameters of the logistic regression. Note that $\beta _0$, the intercept of the regression, refers to a side bias, i.e., if someone preferentially chooses the left or the right arm without any further knowledge.

We implemented all models as hierarchical Bayesian models. We changed the softmax decision rule, which has shown to be the best account for human choices in the Restless bandit task^3,7^, to an upper-confidence bound decision rule. These two adaptations did not change the pattern of group-level effects. The new implementation, however, led to three desired effects. First, it improved the parameter values of the individual effects as compared to the originally proposed models via shrinkage. In particular, it reduced the effect of parameter outliers when fitting individual participant data using Maximum Likelihood estimation, likely due to parameter trade-offs (see Figure S1 in the SI). Second, the model parameters had a higher reliability. Third, it improved the models’ predictive accuracy on held-out data (see Figure S1 in the SI).

To fit the logistic regression choice models in the Horizon task and the Two-armed bandit, we used the brms package^85^ in R^86^. A comparison showed that neither the group-level nor the subject-level parameter estimates were changed in our hierarchical Bayesian implementation compared to the originally proposed subject-level implementation (see Wilson et al.^4^, Figure S2 in the SI). However, both the reliability and the out-of-sample prediction improved (see SI Table S3 and S4, respectively).

#### Improved models to measure exploration strategies

*First*, we removed random exploration from the model in the Two-armed bandit, thus reducing the number of predictors from three to two but leaving the rest of the model unchanged. This change was motivated by the redundancy in the two strategies as indicated by the high correlation between their respective predictors (on average between .83 to .94 across trials 2–10 in the four conditions; see Fig. S3 and S4 in the SI)^87^, between the recovered parameters ($-.79$ and $-.78$; see Fig. 2), and between the estimates of the two strategies ($-.88$; see Fig. 4). In addition, random exploration in the Two-armed bandit likely captured value-guided variance given its positive correlation with value-guided exploration in the Restless bandit.

*Second*, we only used the data from the long horizon condition to estimate value-guided exploration and directed exploration in the Horizon task. Traditionally, exploration is defined as the change in the respective regression parameter from the short to the long horizon condition. This is problematic because it differs from the definition as the absolute value of the respective parameters in the other two bandit tasks. Difference measures are moreover known to have a stronger contamination by error variance^40,41,88^, which reduces their reliability. As it has been hypothesized and observed that people explore more in the long horizon condition than in the short horizon condition^4,15,16^, we exclusively focused on the first free choice from the former.

*Third*, we computed latent factors of the two remaining exploration strategies to remove unwanted sources of variance (i.e., task-specific variance, error variance, and variance due to parameter trade-offs induced during model fitting^27^) from the strategy estimates. For that we used a confirmatory factor analysis including correlations between residuals of parameters estimated from the same task to capture parameter trade-offs separately in the model^28^. That is, we explicitly tested whether the model parameters in the three tasks measured generalizable aspects of the two strategies. You can find more details on the latent factor modeling in the following.

#### Latent factor modeling

Structural equation modeling (SEM) is a latent-variable approach that attempts to estimate the scores of underlying latent constructs using a set of observable indicator variables by modeling the underlying variance-covariance matrix of all observed variables^31^. Before creating a structural model relating different constructs, it is recommended to create separate measurement models for every measured construct^31^ to test whether the individual constructs are actually measurable. One advantage of SEM compared to principle component analysis (i.e., PCA), is that methods variance, for example variance due task strategies or variance due to parameter trade-offs, can be modeled as correlated residuals in the indicator variables^28^. This approach ensures that variance in model parameters not due to the underlying construct is not propagated forward, for example in a correlation with another measure. We want to highlight here that the common practice of using model parameters from a single task propagates methods variance and error variance, and therefore produces biased correlation estimates. The propagation of methods variance also happens within a PCA approach.

Model interpretation requires an acceptable fit of the model to the data. We refer to the guidelines provided by^89^, which state that an acceptable fit is reflected in a Comparative Fit Index (CFI) > .9, and a root mean squared error of approximation (RMSEA) < .08. Because value guided exploration traded off heavily with random exploration in the two-armed bandit, and because there were inconsistent relationships between the value-guided exploration parameters in the three tasks using the original models, we focused the latent modeling on the parameters from the improved models. We created measurement models for working memory capacity, value-guided exploration and directed exploration using the respective indicators (for working memory capacity, we used the proportion of responses reported in the correct serial position, first aggregated per set size and then aggregated over set sizes for each participant). These measurement models were just identifiable with df = 0 and the respective factor loadings are displayed in Fig. 3 .

We then created two structural models, one model with two latent exploration strategies (i.e., value-guided and directed), and one model with only one latent exploration strategy (i.e., all six indicators loading on the same construct). The former model did not converge using the data from the first session. We therefore fit the same two models to the data from the second session (CFI = 0.989, RMSEA = 0.045 for the two-strategy model, and CFI = 0.868, RMSEA = 0.186 for the one-strategy model). Approximating the Bayes factor between the two models with the method provided by^49^ showed that the two-strategy model was clearly preferred.

Because directed exploration was not measurable in the first session, we fitted a structural model only including value-guided exploration and working memory capacity on the data from both sessions to calculate the reliability of the two factors. This model fitted well (CFI = 0.971, RMSEA = 0.059) and showed that both latent factors are measurable reliably over a time period of 6 weeks. The respective correlations were .78 and .91 for value-guided exploration and working memory capacity, respectively. Eventually, we fit a model including the two exploration strategies and working memory capacity, and the five questionnaire-based constructs (i.e., openness to new experiences, exploration, positive mood, negative mood, and anxiety/depression) to the data from Session 2. Note that we computed the questionnaire constructs based on compound scores (i.e., not item-level scores) as has been validated and suggested before^35–39^ . The fit of this joint model was acceptable with a CFI of 0.96 and an RMSEA of 0.05. The latent-level correlations based on this final model can be found in Fig. 6.

#### Optimality analyses

We performed several simulations to test whether the tasks motivate participants to explore. For each of the bandit tasks, we analyzed both our reward sets and reward sets from the literature. Specifically, we simulated choices given a range of parameter combinations. In the Horizon task and the Two-Armed bandit, we simulated 10 choices per parameter combination, in the Restless bandit 50 choices, and then evaluated which combination yielded the best rewards. We used the same deterministic reward set for every parameter combination for our own reward sets, since every participant deterministically saw the same rewards given they made the same choices. We compared this to two reward sets from the literature: The dataset by Zajkowski et al.^12^ for the Horizon task and the dataset by Fan et al.^13^ for the Two-armed bandit. Since reward sets were not fixed in those studies, we randomly sampled a participant on each iteration and simulated choices using that participant’s reward set. For the Restless bandit, in the absence of a suitable public dataset, we simulated random rewards for every participant using our reward generating process with a different seed value (same process as in Daw et al.^3^, see **Bandit Tasks**).

Additionally, we wanted to explicitly test whether participants would benefit from selecting the less explored arm in the long Horizon condition of the Horizon task. We therefore simulated choices for both of our reward sets as well as for all participants in Zajkowski et al.^12^ and deterministically had the model choose the less explored arm in the unequal information, long Horizon condition. We then tested on how many rounds the model’s estimate of which arm was better had changed after observing the reward from that first free choice. Finally, we tested for how many of these rounds the model’s estimate of which arm was better had actually improved, as opposed to changing into the incorrect direction.

## Supplementary Information

Supplementary Information.

## Data Availability

All data is publicly available through the Open Science Framework and can be accessed through the following link: https://osf.io/ra7su/.
