# Supplementary material for: Model-based exploration is measurable across tasks but not linked to personality and psychiatric assessments
Source: Sci Rep. 2025 Jul 28;15:27479. doi: 10.1038/s41598-025-09152-2 (PMC12304400; doi:10.1038/s41598-025-09152-2)
Supplement: Supplementary file 1 — Supplementary Information. [file 41598_2025_9152_MOESM1_ESM.pdf]

Supplementary information for “Model-based exploration is measurable across tasks but not linked to personality and psychiatric assessments”

Tables

Table 1. Model comparison for the two measurement models representing exploration as one factor or as two factors (i.e., value-guided and directed).

Model	χ^2	df	p	CFI	RMSEA [90% CI]	SRMR	AIC	BIC
2 Factors	5.59	5	0.35	1	0.03 [0, 0.11]	0.02	2722	2772
1 Factor	42.3	6	0	0.87	0.19 [0.14, 0.24]	0.08	2756	2804

Table 2. Fit indices for the measurement model based on the questionnaire scores.

Model	χ^2	df	p	CFI	RMSEA [90% CI]	SRMR	AIC	BIC
4 Factors	12.36	10	0.26	0.995	0.037 [0, 0.09]	0.03	2008	2065

Table 3. Test-retest reliability of hierarchical vs subject-level estimates of the parameters in the Horizon task

method	predictor	horizon	correlation
hierarchical	value-guided	long	0.57
hierarchical	value-guided	short	0.52
hierarchical	directed	long	0.17
hierarchical	directed	short	0.51
subject-level	value-guided	long	0.38
subject-level	value-guided	short	0.16
subject-level	directed	long	0.01
subject-level	directed	short	0.16

Table 4. Out-of-sample prediction for the hierarchical and the subject-level implementation of the model in the Horizon task. We predicted session two data using parameters fit on session one data.

method	Horizon	Log Likelihood
hierarchical	short	$-1,988.125$
hierarchical	long	$-2,137.137$
subject-level	short	$-2,884.124$
subject-level	long	$-2,492.672$

Table 5. Retest reliability in the form of ICC3(C,1) and ICC3(A,1) for the model parameters of the original models and the task measures in the three tasks.

Task	Parameter	ICC	Measure	Retest Reliability
Horizon	Directed	Agreement	Parameter	0.273
Horizon	Value-Guided	Agreement	Parameter	0.0940
Horizon	p(switch)	Agreement	Task Measure	0.686
Horizon	p(optimal)	Agreement	Task Measure	0.694
Horizon	Regret	Agreement	Task Measure	0.701
Horizon	Directed	Consistency	Parameter	0.300
Horizon	Value-Guided	Consistency	Parameter	0.105
Horizon	p(switch)	Consistency	Task Measure	0.686
Horizon	p(optimal)	Consistency	Task Measure	0.694
Horizon	Regret	Consistency	Task Measure	0.701
Restless	Directed	Agreement	Parameter	0.514
Restless	Value-Guided	Agreement	Parameter	0.442
Restless	p(switch)	Agreement	Task Measure	0.557
Restless	p(optimal)	Agreement	Task Measure	0.203
Restless	Regret	Agreement	Task Measure	0.250
Restless	Directed	Consistency	Parameter	0.513
Restless	Value-Guided	Consistency	Parameter	0.471
Restless	p(switch)	Consistency	Task Measure	0.562
Restless	p(optimal)	Consistency	Task Measure	0.206
Restless	Regret	Consistency	Task Measure	0.259
Two-Armed	Directed	Agreement	Parameter	0.505
Two-Armed	Value-Guided	Agreement	Parameter	0.357
Two-Armed	Random	Agreement	Parameter	0.477
Two-Armed	p(switch)	Agreement	Task Measure	0.613
Two-Armed	p(optimal)	Agreement	Task Measure	0.391
Two-Armed	Regret	Agreement	Task Measure	0.370
Two-Armed	Directed	Consistency	Parameter	0.511
Two-Armed	Value-Guided	Consistency	Parameter	0.473
Two-Armed	Random	Consistency	Parameter	0.484
Two-Armed	p(switch)	Consistency	Task Measure	0.621
Two-Armed	p(optimal)	Consistency	Task Measure	0.442
Two-Armed	Regret	Consistency	Task Measure	0.372

Table 6. Retest reliability in the form of ICC3(C,1) and ICC3(A,1) for the model parameters of the improved models.

Task	Parameter	ICC	Measure	Value
Horizon	Value-Guided	Agreement	Parameter	0.570
Horizon	Directed	Agreement	Parameter	0.130
Horizon	Value-Guided	Consistency	Parameter	0.610
Horizon	Directed	Consistency	Parameter	0.183
Two-Armed	Value-Guided	Agreement	Parameter	0.312
Two-Armed	Directed	Agreement	Parameter	0.498
Two-Armed	Value-Guided	Consistency	Parameter	0.413
Two-Armed	Directed	Consistency	Parameter	0.497

Table 7. Retest reliability in the form of ICC3(C,1) and ICC3(A,1) for the working memory and questionnaire measures.

Domain	Measure	Retest Reliability	ICC
Questionnaire	Openness	0.879	Consistency
Questionnaire	Openness	0.879	Agreement
Questionnaire	Exploration	0.736	Consistency
Questionnaire	Exploration	0.737	Agreement
Questionnaire	Negative Mood	0.754	Consistency
Questionnaire	Negative Mood	0.753	Agreement
Questionnaire	Positive Mood	0.823	Consistency
Questionnaire	Positive Mood	0.824	Agreement
Questionnaire	Depression	0.884	Consistency
Questionnaire	Depression	0.885	Agreement
Questionnaire	Anxiety Cog.	0.888	Consistency
Questionnaire	Anxiety Cog.	0.888	Agreement
Questionnaire	Anxiety Som.	0.754	Consistency
Questionnaire	Anxiety Som.	0.747	Agreement
WM	Operation Span	0.757	Consistency
WM	Operation Span	0.742	Agreement
WM	Symmetry Span	0.769	Consistency
WM	Symmetry Span	0.738	Agreement
WM	WM Updating	0.723	Consistency
WM	WM Updating	0.668	Agreement

Table 8. Correlations between original model parameters that we simulated from and recovered parameters using our implementation. Directed exploration = cor(information bonus, weight of difference in information), random exploration = cor(inverse choice temperature, weight of difference in means), side bias = cor(side bias, Intercept of regression)

Horizon	directed exploration	random exploration	side bias
short	0.75	0.82	0.86
long	0.81	0.82	0.86

Figures

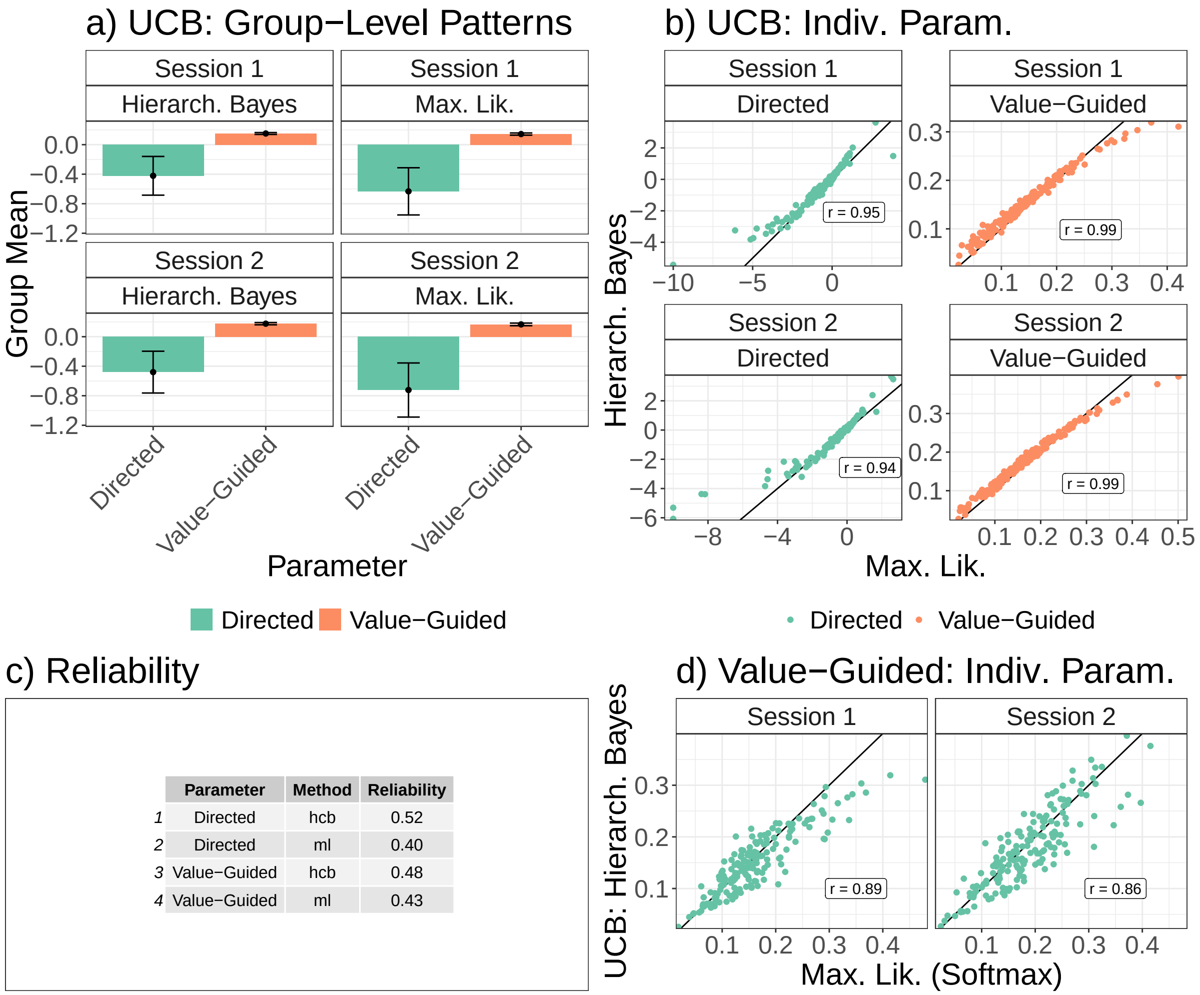

	Parameter	Method	Reliability
1	Directed	hcb	0.52
2	Directed	ml	0.40
3	Value−Guided	hcb	0.48
4	Value−Guided	ml	0.43

Figure 1. *Comparing Maximum Likelihood models and a Hierarchical Bayesian model in the restless bandit task.* Panel a) shows that the group-level pattern was the same between the two methods. Panel b) shows that the correlation of the model parameters between the two methods was extremely high, and that the hierarchical Bayesian method pulled outliers, as calculated with ML, closer to the overall group mean (i.e., shrinkage). Panel c) shows that the reliabilities of the model parameters were larger in the hierarchical Bayesian method than in the ML method. Panel d) shows a high correlation between value-guided exploration in the softmax model (i.e., only value-guided exploration) and in the UCB model (i.e., value-guided and directed exploration)

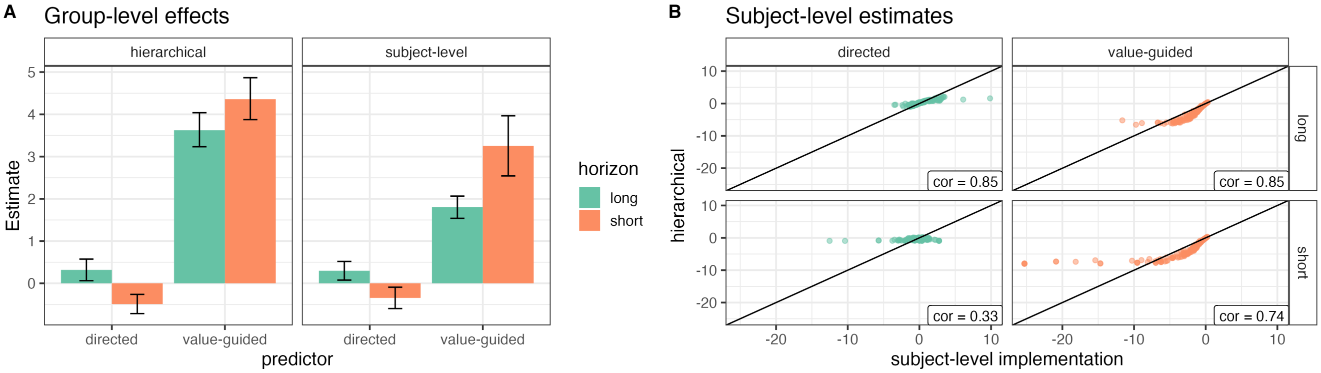

Comparing Maximum Likelihood models and a Hierarchical Bayesian model in the Horizon task.

Figure 2. **A:** The patterns of group-level effects remain qualitatively unchanged when replacing the classic subject-level modeling approach with a hierarchical Bayesian approach. **B:** Similarly, the parameter estimates from the classic subject-level modeling approach are highly correlated with the parameter estimates from the hierarchical Bayesian approach. The latter does however yield a much narrower distribution of estimates by avoiding extreme outliers.

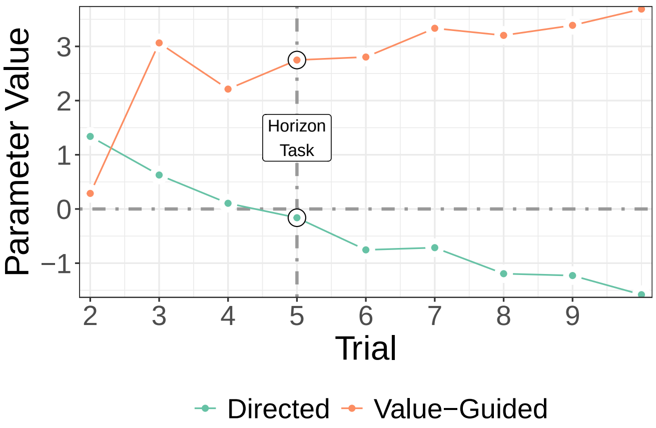

Figure 3. Parameter values for value-guided and directed exploration plotted over trials when a hierarchical model is separately fitted to the choices in trials 2 - 10 in the Two-armed bandit task. Trial 5 in the Two-armed bandit reflects the first free choice in the Horizon task.

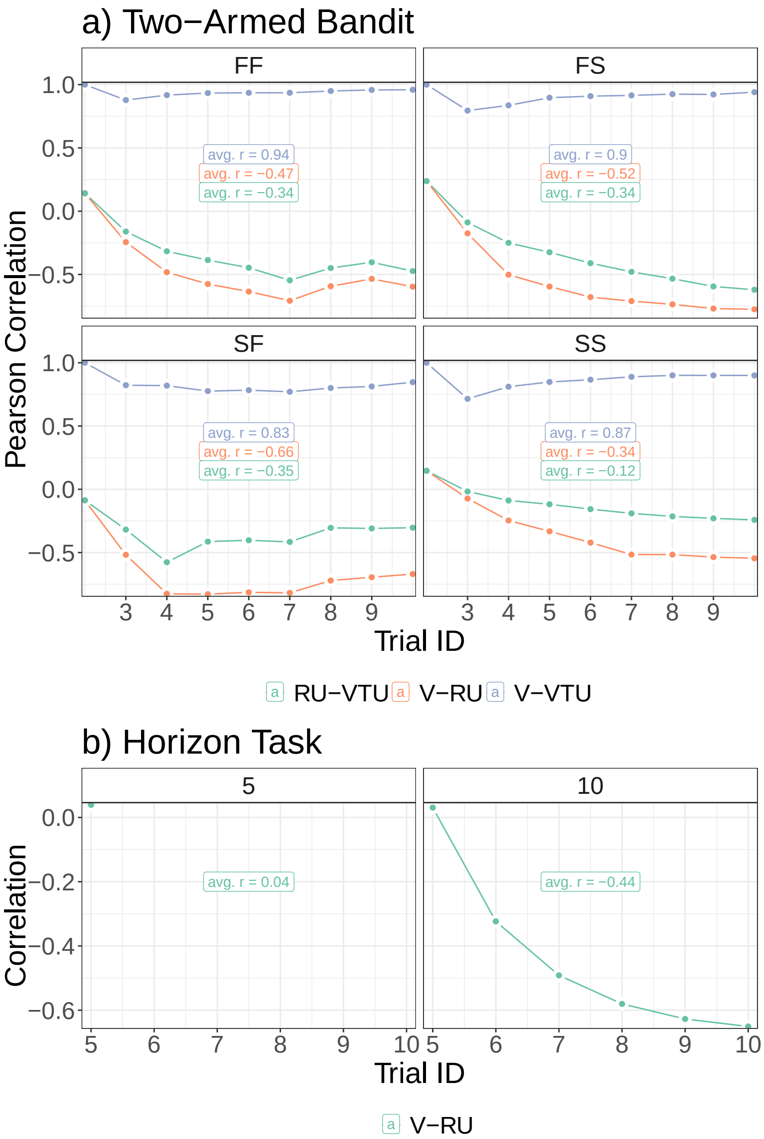

Figure 4. a) Correlations between differences in expected value (V), differences in uncertainties (RU), and differences in expected value divided by total uncertainty (VTU) in the Two-armed bandit tasks[1]. b) Correlations between value-guided exploration (V) and directed exploration (RU) in the Horizon task. The correlation between value-guided exploration and directed exploration in the Restless bandit task was -.19.

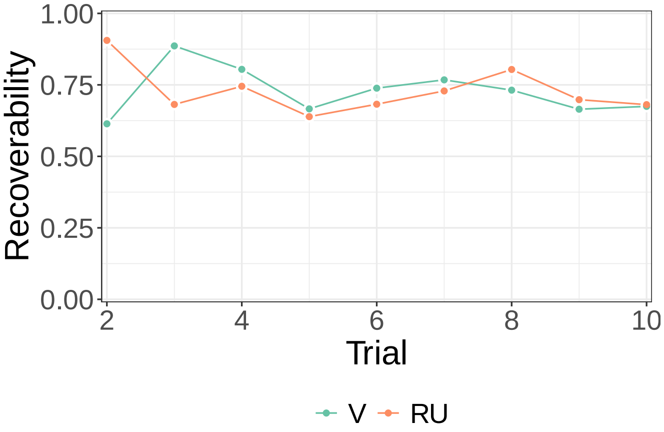

Figure 5. Recoverability of value-guided and directed exploration when analyzing choices 2-10 in the Two-armed bandit separately. On average, the MAP estimate for each participant, was recoverable with a correlation of .73 and .73 for value-guided exploration and directed exploration, respectively.

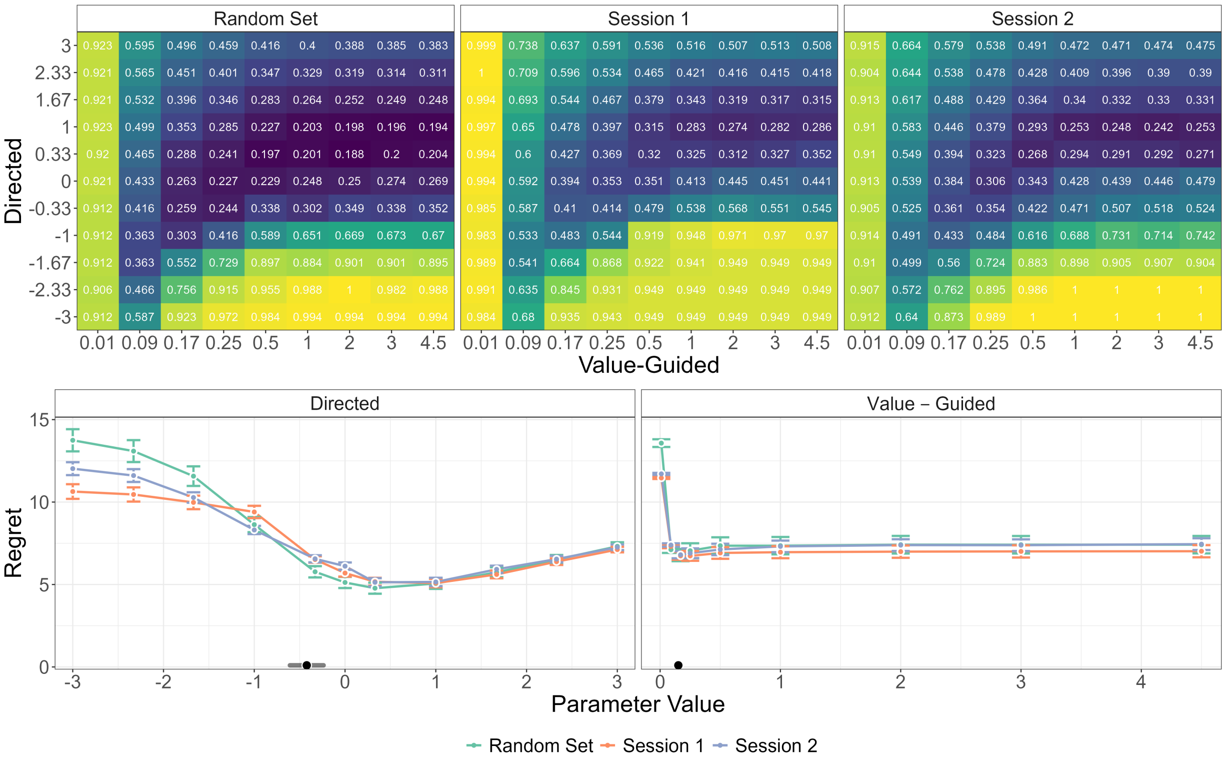

Figure 6. Average regret by agents with a different combination of parameter combinations in the four-armed restless bandit task. In the top row, the average regret for each parameter combination was divided by the maximum average regret across all possible parameter combinations. In the bottom row, the average regret is computed for each value of value-guided and directed exploration, marginalizing over all values of the not considered strategy. Note: Error bars represent 95% confidence intervals. The black point and the gray errorbar represent the mean and the 95% confidence interval of the by-participant maximum a posteriori (MAP) estimates from the first session.

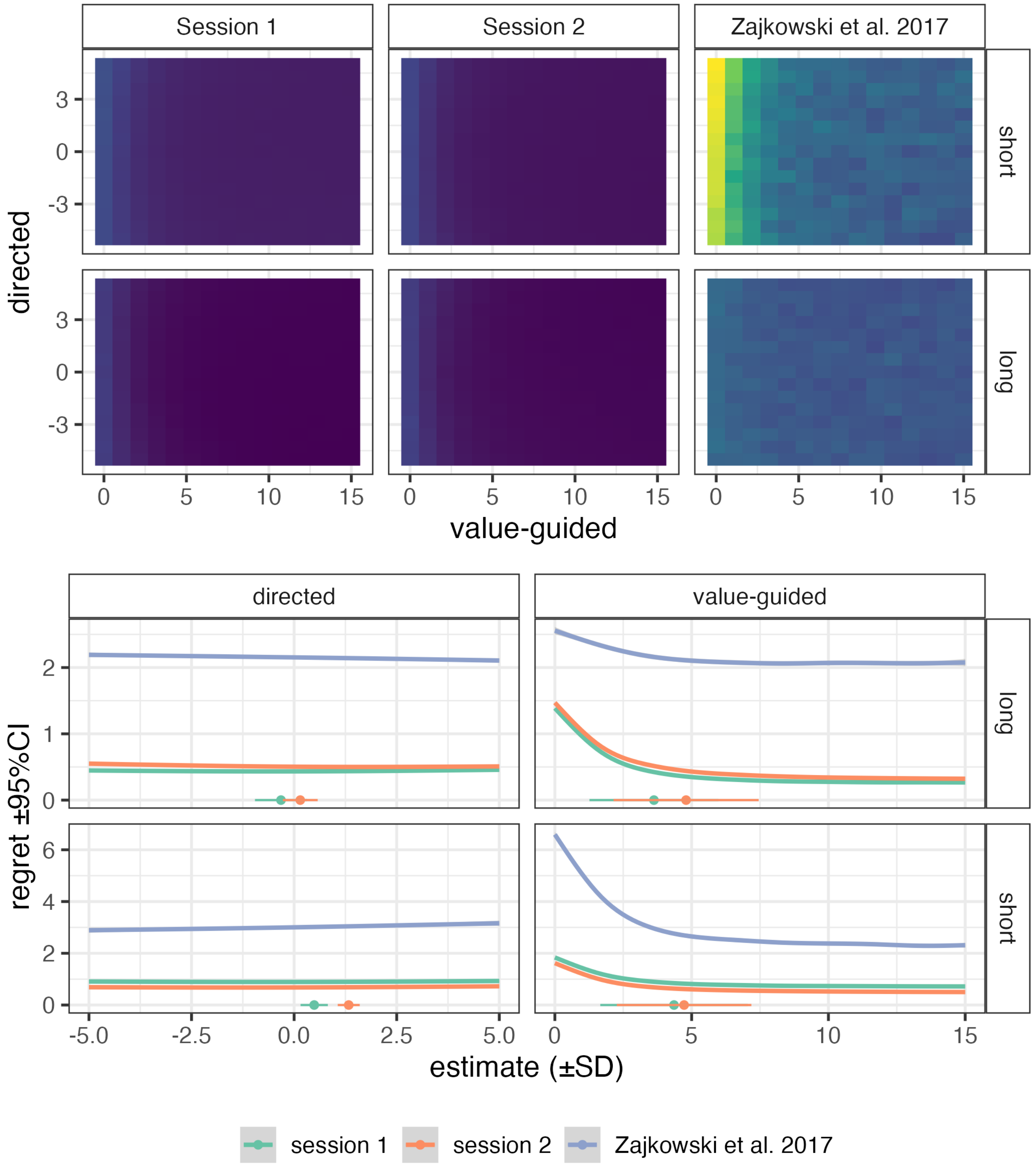

Figure 7. Average regret on a trial (optimal reward - received reward) on the Horizon task for different parameter estimates. We compare both of our reward sets (session 1 an session 2) to a reward set from Zjakowski et al.[2]. The top figure shows the average regret for each combination of possible levels of directed and random exploration (lighter colors indicate higher regret, i.e. worse performance). In the bottom figure, regret for each estimate is computed by averaging over all values of the not considered strategy as well as over a range of possible intercepts. For our own reward sets we also plot participants' parameter estimates in the bottom figure. The rows in both figures represent the long versus the short horizon condition.

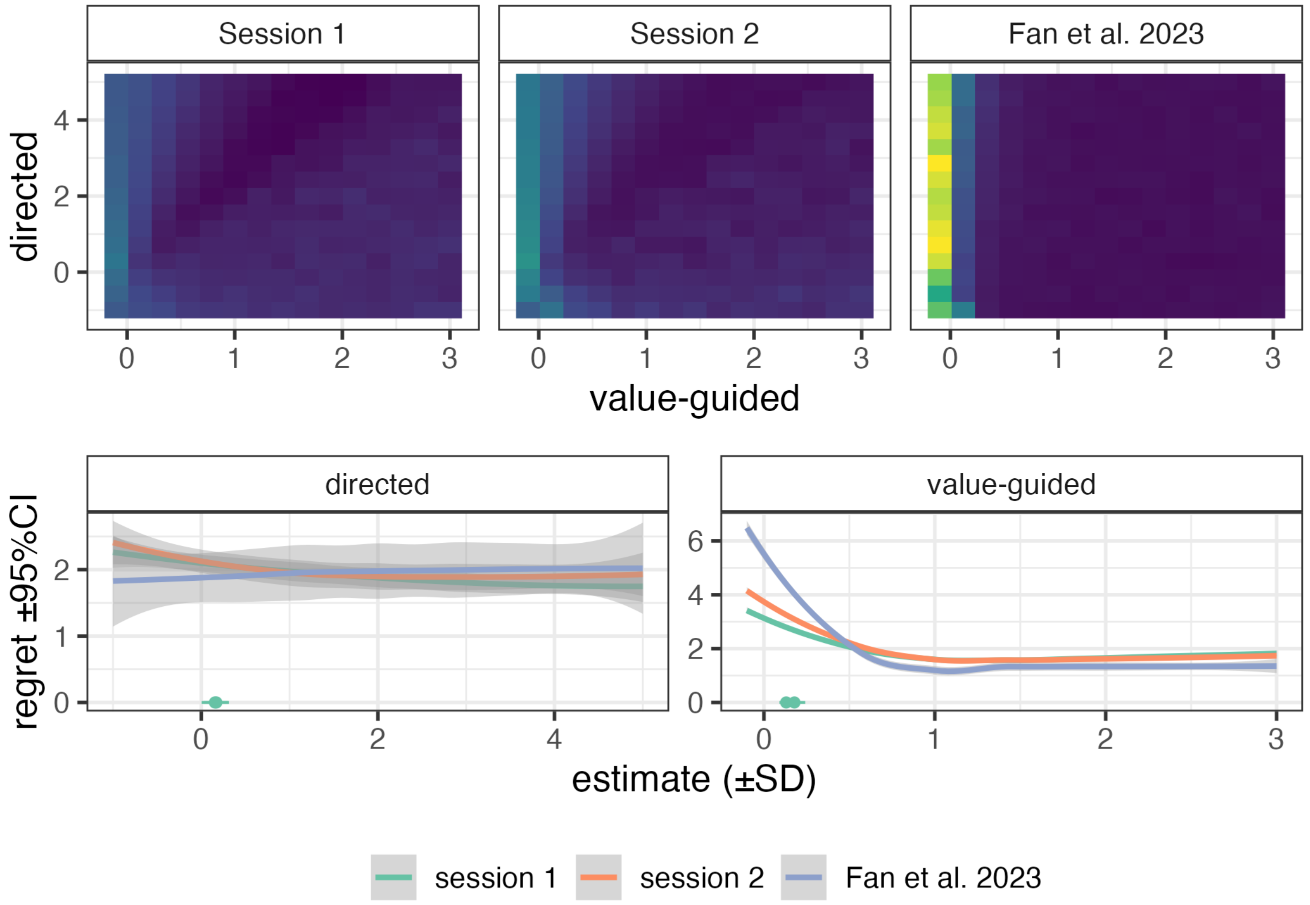

Figure 8. Average regret on a trial (optimal reward - received reward) on the Two-armed bandit for different parameter estimates. We compare both of our reward sets (session 1 an session 2) to a reward set from Fan et al.[3]. The top figure shows the average regret for each combination of possible levels of directed and random exploration (lighter colors indicate higher regret, i.e. worse performance). In the bottom figure, regret for each estimate is computed by averaging over all values of the not considered strategy. For our own reward sets we also plot participants' parameter estimates in the bottom figure.

References

1. Gershman, S. J. Deconstructing the human algorithms for exploration. *Cognition* **173**, 34–42, DOI: 10.1016/j.cognition.2017.12.014 (2018).
2. Zajkowski, W. K., Kossut, M. & Wilson, R. C. A causal role for right frontopolar cortex in directed, but not random, exploration. *eLife* **6**, e27430, DOI: 10.7554/eLife.27430 (2017). Publisher: eLife Sciences Publications, Ltd.
3. Fan, H., Gershman, S. J. & Phelps, E. A. Trait somatic anxiety is associated with reduced directed exploration and underestimation of uncertainty. *Nat. Hum. Behav.* **7**, 102–113, DOI: 10.1038/s41562-022-01455-y (2023). Number: 1 Publisher: Nature Publishing Group.
